# Identification and validation of a machine learning model of complete response to radiation in rectal cancer reveals immune infiltrate and TGFβ as key predictors

**DOI:** 10.1016/j.ebiom.2024.105228

**Published:** 2024-07-16

**Authors:** Enric Domingo, Sanjay Rathee, Andrew Blake, Leslie Samuel, Graeme Murray, David Sebag-Montefiore, Simon Gollins, Nicholas West, Rubina Begum, Susan Richman, Phil Quirke, Keara Redmond, Aikaterini Chatzipli, Alessandro Barberis, Sylvana Hassanieh, Umair Mahmood, Michael Youdell, Ultan McDermott, Viktor Koelzer, Simon Leedham, Ian Tomlinson, Philip Dunne, S:CORT consortium, Andrew Blake, Francesca Buffa, Enric Domingo, Geoffrey Higgins, Christopher Holmes, Viktor Koelzer, Simon Leedham, Timothy Maughan, Gillies McKenna, James Robineau, Ian Tomlinson, Michael Youdell, Philip Quirke, Susan Richman, David Sebag-Montefiore, Matthew Seymour, Nicholas West, Philip Dunne, Richard Kennedy, Mark Lawler, Keara Redmond, Manuel Salto-Tellez, Peter Campbell, Aikaterini Chatzipli, Claire Hardy, Ultan McDermott, Simon Bach, Andrew Beggs, Jean-Baptiste Cazier, Gary Middleton, Dion Morton, Celina Whalley, Louise Brown, Richard Kaplan, Graeme Murray, Richard Wilson, Richard Adams, Richard Sullivan, Leslie Samuel, Paul Harkin, Steven Walker, Jim Hill, Chieh-Hsi Wu, Dennis Horgan, Francesca M. Buffa, Timothy S. Maughan

**Affiliations:** a Department of Oncology, Medical Sciences Division, University of Oxford, Old Road Campus Research Building, Roosevelt Drive, Oxford, OX3 7DQ, UK; b School of Medicine, Medical Sciences and Nutrition, University of Aberdeen, Foresterhill, Aberdeen, AB25 2ZD, UK; c Leeds Institute of Medical Research, University of Leeds, LS9 7TF, UK; d North Wales Cancer Treatment Centre, Besti Cadwaladr University Health Board, Bodelwyddan, Denbighshire, LL18 5UJ, UK; e Cancer Research & University College London Clinica Trial Unit, London, United Kingdom; f The Patrick G Johnston Centre for Cancer Research, Queens University Belfast, Belfast, BT7 9AE, UK; g Wellcome Sanger Institute, Hinxton, Cambridge, CB10 1RQ, UK; h Department of Pathology and Molecular Pathology, University Hospital Zurich, University of Zurich, Zurich, Switzerland; i Department of Oncology and Nuffield Department of Medicine, University of Oxford, Oxford, United Kingdom; j Wellcome Trust Centre for Human Genetics, Nuffield Department of Medicine, University of Oxford, UK; k Department of Computing Sciences, Bocconi University, Bocconi Institute for Data Science and Analytics (BIDSA), Milano, Italy; l Department of Molecular and Clinical Cancer Medicine, University of Liverpool, Liverpool, UK

**Keywords:** Rectal neoplasms, Radiotherapy, Precision medicine, Prediction, TGFβ, Immune response, Genes

## Abstract

**Background:**

It is uncertain which biological features underpin the response of rectal cancer (RC) to radiotherapy. No biomarker is currently in clinical use to select patients for treatment modifications.

**Methods:**

We identified two cohorts of patients (total N = 249) with RC treated with neoadjuvant radiotherapy (45Gy/25) plus fluoropyrimidine. This discovery set included 57 cases with pathological complete response (pCR) to chemoradiotherapy (23%). Pre-treatment cancer biopsies were assessed using transcriptome-wide mRNA expression and targeted DNA sequencing for copy number and driver mutations. Biological candidate and machine learning (ML) approaches were used to identify predictors of pCR to radiotherapy independent of tumour stage. Findings were assessed in 107 cases from an independent validation set (GSE87211).

**Findings:**

Three gene expression sets showed significant independent associations with pCR: Fibroblast-TGFβ Response Signature (F-TBRS) with radioresistance; and cytotoxic lymphocyte (CL) expression signature and consensus molecular subtype CMS1 with radiosensitivity. These associations were replicated in the validation cohort. In parallel, a gradient boosting machine model comprising the expression of 33 genes generated in the discovery cohort showed high performance in GSE87211 with 90% sensitivity, 86% specificity. Biological and ML signatures indicated similar mechanisms underlying radiation response, and showed better AUC and p-values than published transcriptomic signatures of radiation response in RC.

**Interpretation:**

RCs responding completely to chemoradiotherapy (CRT) have biological characteristics of immune response and absence of immune inhibitory TGFβ signalling. These tumours may be identified with a potential biomarker based on a 33 gene expression signature. This could help select patients likely to respond to treatment with a primary radiotherapy approach as for anal cancer. Conversely, those with predicted radioresistance may be candidates for clinical trials evaluating addition of immune-oncology agents and stromal TGFβ signalling inhibition.

**Funding:**

The Stratification in Colorectal Cancer Consortium (S:CORT) was funded by the 10.13039/501100000265Medical Research Council and 10.13039/501100000289Cancer Research UK (MR/M016587/1).

**Research in context**

**Evidence before this study**

Patients with RC are usually treated with radiotherapy as part of neoadjuvant therapy although they show a wide range of responses. Currently there are no clinical or molecular biomarkers implemented in the clinic to select which patients benefit or not from such treatment. We searched PubMed with the terms “rectal”, “radiotherapy”, and “expression”, for articles published up to Oct 2, 2020. Nine studies reported a gene expression RNA signature for response to CRT in primary RC using pre-treatment biopsies. However, they had important limitations, notably low statistical power, with the largest discovery set being only 77 cases, and a missing validation set in 3 cases. Furthermore, most studies had an heterogeneous clinical setting including diverse regimens of chemotherapy combined with conventionally fractionated radiotherapy.

**Added value of this study**

Our study used a discovery set of 249 cases, drawn from one large case series and the control arm of a national clinical trial. Multi-omic profiling was undertaken by a multi-institution consortium using state of the art analytical tools. Advanced ML algorithms and methodology were used to detect previously defined gene sets associated with pCR. The findings were validated in an independent, external dataset of 107 cases and the predictive performance compared against all other published signatures. We reveal clear biological features (immune activity) underpinning response to radiation and define predictive biomarkers of pCR with excellent performance.

**Implications of all the available evidence**

We find that the stromal and immune cell compartments within tumours are key determinants of pCR to CRT in RC. Targeting these compartments provide the basis for novel studies using improved patient selection and combination treatments, which may be able to improve radiation response.

## Introduction

Rectal Cancer (RC) accounts for 30% of the total incidence of colorectal cancer (CRC) and radiotherapy is used as part of the primary treatment of 40% patients with RC.^1^ Pre-operative staging with MRI of the pelvis identifies patients with locally advanced RCs, in whom the surgical resection margin is threatened or involved, and in these people neoadjuvant treatment including chemoradiation or short course radiotherapy with pre-operative chemotherapy is indicated, with the intention of maximising the likelihood of achieving an R0 resection.^2^ Neoadjuvant therapy also has an emerging role in the non-operative management of RC in patients who are reluctant or unfit to undergo radical surgery, or for those in whom pCR is observed in the 6–12 weeks following completion of CRT. In these patients a watch and wait policy may be undertaken and a recent meta-analysis shows increased local recurrence rates but long term outcomes comparable in clinical complete responders to total mesorectal excision.^3^

Prediction of response to CRT in RC has not been possible to date despite a number of small scale attempts to define predictive biomarkers from the transcriptome.^4^, ^5^, ^6^, ^7^, ^8^, ^9^, ^10^, ^11^, ^12^ This is plausibly owing to a number of limitations in such studies such as different chemotherapy combinations with radiotherapy, unmatched endpoints mostly based on different cut points to call response, lack of correction for stage, critically low statistical power, and absence of a validation cohort (Supplementary Table S1). Notably, reported biological insights from such studies are few and inconsistent. The lack of biomarkers to predict pCR to neoadjuvant radiotherapy is a clinical unmet need. Such a stratifier could enable a proportion of patients with high likelihood of achieving pCR to be offered definitive, radiation based treatment like in anal cancer, enabling a significant number of patients to be treated without surgery and avoid a stoma. In contrast, identification of those with poor response to CRT, who also have a higher rate of distant metastases,^13^ could spare them the toxic effects and inconveniences of such treatment. It may also enable selection of patients for suitably modulated first line therapy designed to target the underlying biology driving radioresistance and metastasis.

In this study from the Stratification in COloRectTal cancer (S:CORT) consortium, we aimed to identify the biological basis of complete response to radiotherapy in patients with RC and to derive a predictive biomarker. We have undertaken multi-omic profiling of 249 pre-radiotherapy biopsies from two cohorts of patients with RC, comprising transcriptome-wide mRNA expression, mutations in 80 CRC driver genes and genome-wide copy number alterations. Using multiple regression and ML approaches we identify and validate biological predictors of pCR to radiotherapy in RC with a level of performance that suggests clinical utility.

## Methods

### Clinical cohorts

Two independent cohorts of pattients with RC were included in our discovery set: 125 cases from a sequential cohort from the Aberdeen area in the UK (‘Grampian cohort’) and 124 cases from the control arm of the UK multicentre Aristotle clinical trial which compared the efficacy of standard CRT with or without irinotecan (ISRCTN09351447). Patients identified received ‘standard CRT’ comprising pelvic irradiation (45–50.4Gy in 25 fractions over 5 weeks) with capecitabine 900 mg/m^2^ bd days Monday to Friday, throughout radiotherapy (Supplementary Figure S1a and b, Supplementary Methods) based on a threatened or involved circumferential rectal fascia on pre-treatment MRI scan. Sex was included as a covariable. Validation testing was performed on 107 cases from a publicly available RC cohort (GSE87211)^14^ with transcriptomic data from pre-treatment biopsies, selected to have been treated with similar neoadjuvant regimen (50.4Gy in 28 fractions with infusional 5-fluorouracil alone) expected to have equivalent biological effects. Full details for the 3 cohorts are available in Supplementary Methods. The primary endpoint in all cases was pCR after CRT as assessed by specialist pathologist (GIM for Grampian and NPW for Aristotle).

### Profiling

All clinical samples assessed were taken from Formalin Fixed Paraffin Embedded (FFPE) RC biopsies from patients before commencement of CRT given as neoadjuvant therapy for RC management. After assessment of haematoxylin and eosin-stained sections, areas of tumour were macrodissected and nucleic acids were extracted using standard protocols. A specifically designed panel of RNA baits (Agilent SureSelect) enabled capture and sequencing of all exons of 80 CRC driver genes, 66 regions of recurrent copy number gains/losses, 960 reference SNPs distributed across the genome (allowing low resolution copy number estimations) and 123 regions for microsatellite instability (MSI). Samples showing neutral copy number calls in >20% of the length of all chromosomes combined were classified negative for Chromosomal Instability (CIN), otherwise as positive.^15^ RNA expression profiling used the 24,441 genes/110,961 probesets ALMAC/Affymetrix XCEL microarrays (Supplementary Methods).

### Statistics

Twenty pre-defined, hypothesis-based candidate gene sets, pathways or molecular classifiers for response to radiotherapy were selected by an expert panel of researchers and tested for association with pCR using logistic regression analyses (Supplementary Table S2, Supplementary Methods).

In a second, hypothesis-free analysis, our aim was to build a machine learning (ML) model to predict pCR from the transcriptomic data. A pipeline consisting of five steps was established and applied using 12 different modelling approaches: Quality check, Pre-processing, including correction of class imbalance, differentially expressed genes (DEG) selection, Decision making genes (DMG) selection, Training to build a model (ML) from the discovery cohort as previously described.^16^ The best performing ML model was tested on the full GSE87211 validation cohort after appropriate batch correction to derive sensitivity, specificity, and area under curve. Additional models were also tested. The complete methodology is provided in Supplementary Methods.

Additionally, a literature search identified 9 reported signatures for prediction of response to radiotherapy in RC (Supplementary Table S1) which were also tested in the validation cohort.

### Ethics

The S:CORT consortium including this specific analysis was reviewed and approved by the South Cambs Research Ethics committee (REC ref 15/EE/0241; IRAS reference 169363). All patients provided written informed consent for further research to be undertaken on samples.

### Role of funders

The Stratification in Colorectal Cancer Consortium (S:CORT) was funded by the Medical Research Council and Cancer Research UK. The funders played no role in the study design, data collection, data analyses performed, interpretation or writing of the report.

## Results

### Clinical and molecular profiles

**Fig. 1:**
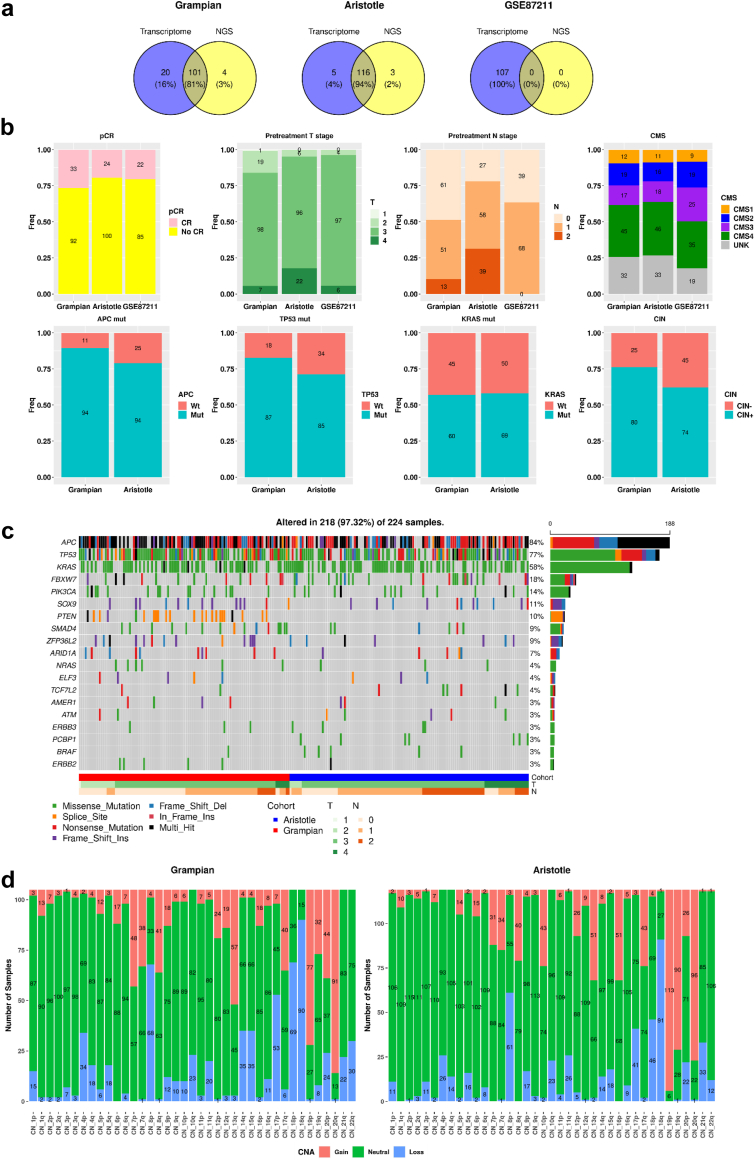
**Clinical and molecular data in Grampian, Aristotle and GSE87211**. a. Overlap of samples profiled for RNA and DNA platforms. b. Comparison of the main clinical and molecular profiles by cohort. c. Most common driver mutations by gene. d. Copy number alterations by chromosome arm. (note RNA data only available from GSE87211).

Most selected samples (87%) from both Grampian (N = 125) and Aristotle (N = 124) were successfully profiled for both transcriptome and targeted NGS, with remaining cases profiled for one of them (Fig. 1a, Supplementary Figure S1a and b). The frequency of pCR in Grampian, Aristotle and GSE87211 cohorts was 26%, 19% and 21% respectively. Both pretreatment T and N stage showed some variation (Fig. 1b, Supplementary Table S3) while the frequencies of the main molecular profiles were within expected ranges, albeit different for some genetic variables such as *APC* and *TP53* mutations and CIN that were more common in Grampian than Aristotle (Fig. 1c and d, Supplementary Figure S2a Supplementary Table S3). Summaries of the most common driver mutations, copy number alterations at chromosome level and transcriptome signatures are shown in Fig. 1 and Supplementary Figure S2.

### Hypothesis-based analysis

**Fig. 2:**
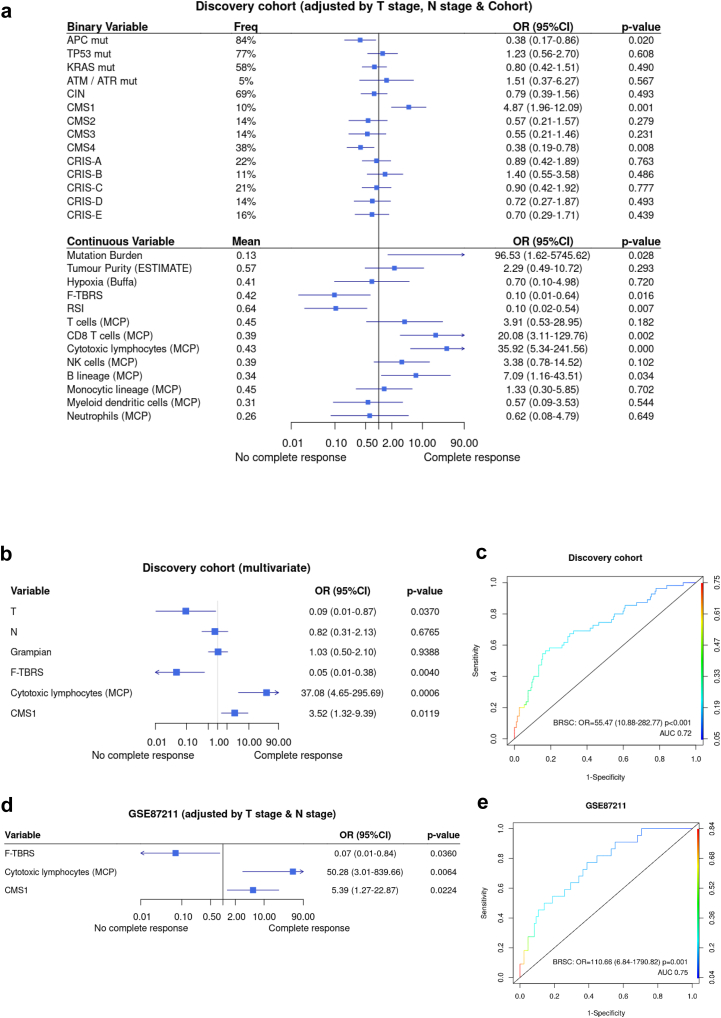
**Candidate analysis**. a. Univariable regression adjusted for T and N stage for prediction of pCR in candidate biological features in discovery cohort (Grampian and Aristotle combined). b. Multivariable model adjusted for T and N stage after stepwise backwards regression in discovery cohort. c. ROC curve applying the 3 variables (F-TBRS, Cytotoxic lymphocytes and CMS1) as one compound variable in discovery cohort. d. Univariable model of the 3 variables (F-TBRS, Cytotoxic lymphocytes and CMS1) adjusted for T and N stage in GSE87211. e. ROC curve applying the 3 BRSC variables combined in GSE87211.

To obtain robust predictive models, Grampian and Aristotle cohorts were merged and relabelled as Discovery cohort (Supplementary Figure S5). Then, logistic regression adjusted by cohort and baseline T and N stage was performed to identify predictors of pCR on 20 prespecified biological candidates (Supplementary Table S2) from our multiomic data. Nine features were significant (Fig. 2a): five associated positively with pCR were all indicators of immune cell abundance (CMS1, mutation burden, CD8 Tcells, cytotoxic lymphocytes and B lineage (by MCP)) and 4 negatively associated (APC mutation, CMS4, F-TBRS and RSI (radiosensitivity index)). After multivariable backward stepwise regression, only three remained independently significant F-TBRS (OR 0.05, p 0.004), cytotoxic lymphocytes (OR 37.08, p 0.0006) and CMS1 (OR 3.52, p 0.012) (Fig. 2b). This final model, which we have labelled as Biological RadioSensitivity Classifier (BRSC), in a ROC curve predicted pCR with AUC of 72% in the discovery set (Fig. 2c). BRSC performance was reproducible in the validation set in prediction of pCR (p = 0.001) showing AUC of 75% (Fig. 2d and e). Its addition to a model with T and N stage was highly significant (p = 0.0008, likelihood ratio test). Meta-analysis of these three variables (CMS1, cytotoxic lymphocytes and F-TBRS) in the three tested datasets showed no significant heterogeneity between cohorts (Supplementary Figure S3).

### Analysis of genetic variables

We interrogated other multiomic profiles not tested as candidates. None of the mutations or copy number changes were significant (Supplementary Figure S4).

### ML analysis

**Fig. 3:**
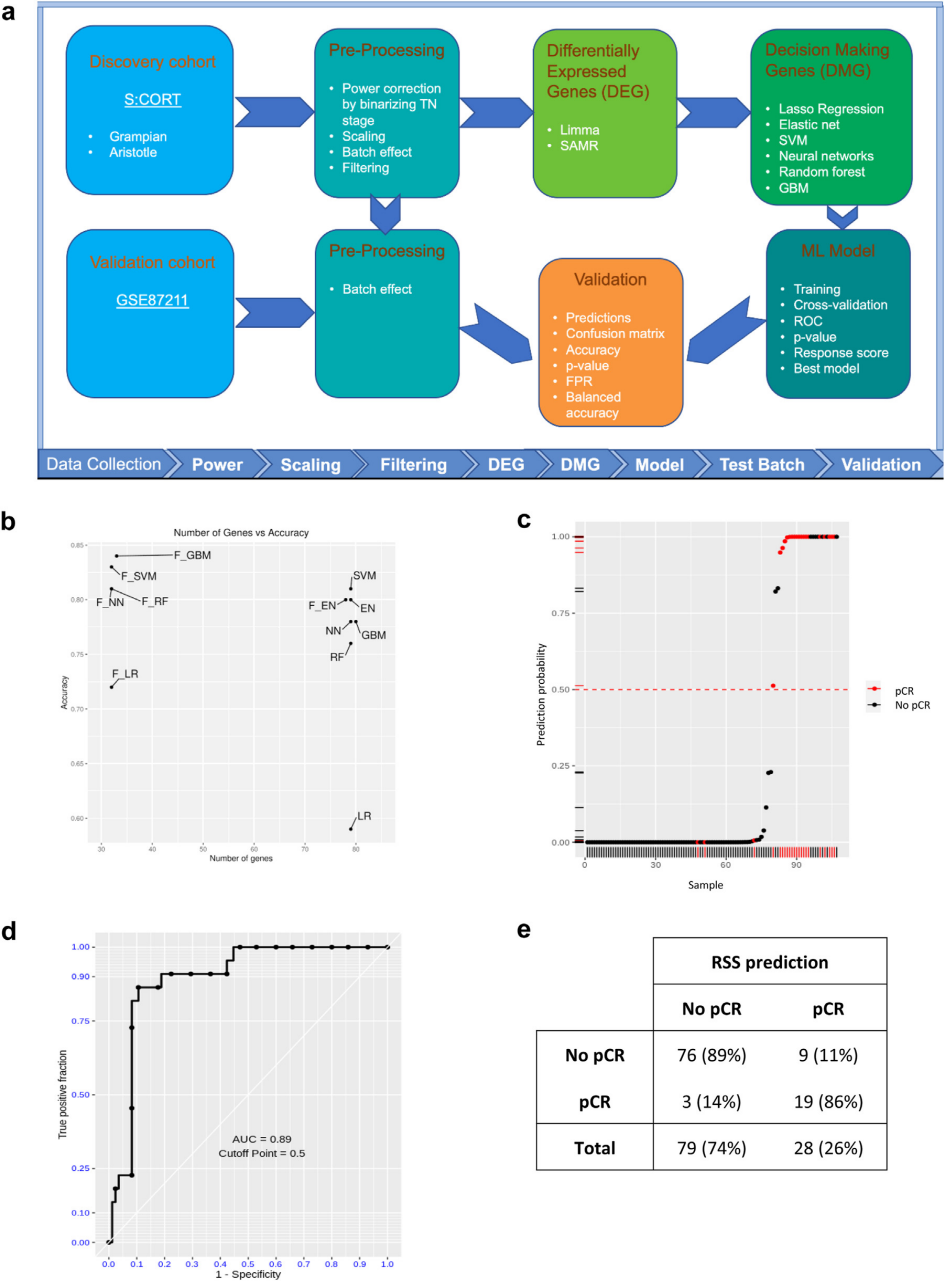
**Machine learning**. a. Analytical pipeline used to derive and validate a new signature to predict pCR. b. 10-fold cross validation accuracy and number of genes for each ML method tested. The one with highest accuracy was selected. EN: elastic net, GBM: gradient boosting machine, LR: lasso regression, NN: neural net; RF: random Forest; SVM: support-vector machine. The prefix F- refers to functional. c. RSS predictive scores from Gradient boosting machine model on GSE87211. d. ROC curve for Gradient boosting machine model on GSE87211. e. Confusion matrix for the new RSS signature in GSE87211.

Using multiple ML methods (Supplementary Methods, Fig. 3a, Supplementary Figure S6) we aimed to develop a locked ML model as recommended by FDA.^16^ We compared their performance in cross-validation, and identified in the discovery cohort the gradient boosting machine as providing the optimal geneset based on highest K-fold cross validation accuracy (84%) with lowest number of genes required (33) (Fig. 3b, Supplementary Table S4). This locked model applied to all GSE87211 showed 89% accuracy and 89% AUC (Fig. 3c and d) with 89% sensitivity and 86% specificity (Fig. 3e), showing excellent ability to differentiate between pCRs and non-pCRs. Similar results were found when using a subset of GSE87211 balanced for pCR (Supplementary Figure S7b–d). We labelled this new geneset as RadioSensitive Signature (RSS). Considering the difference between the training and validation cohorts, batch correction was necessary (Supplementary Figure S7). Based on our research we identified the SVA tool (Combat function) as the most relevant algorithm for batch correction.^17^ This requires some processing of the validation data. In particular, to avoid degrading the pCR signal during the batch correction, pCR data has to be disclosed to the batch correction function to properly merge the discovery and GSE87211 cohorts (full discussion on this is provided in Supplementary Methods). However RSS was also tested for validation without standardisation of the analytical platform by using a version of the validation transcriptome built fully agnostically from outcome. This procedure is suboptimal from a batch correction perspective, but it is an important validation that RSS was still predictive in a setting not disclosing the pCR data (64% accuracy, 74% AUC, p = 0.04) (Supplementary Figure S7e–g).

Finally, we performed a randomized permutation experiment to further test our geneset. An empirical null was generated by randomly picking 1000 gene sets (each containing 33 genes). The AUC for the discovery (Supplementary Figure S7h) & validation cohort (Supplementary Figure S7i and j) estimated using this empirical null was found significant (p-value <0.001, Predictor p-values in linear models).

### BRSC and RSS across grades of clinical response

We have used pCR as a valuable clinical endpoint with a clear biological meaning. We asessed whether RSS might also provide additional granular information for broader response to radiotherapy than specifically for pCR. We checked the distribution of RSS scores across 4 levels of pathological response in the single cohort (Grampian) with these data available and across pathological T stage after treatment in the whole Discovery cohort and GSE87211 (Supplementary Figure S8a–c). pCRs and yT0 cases showed higher levels of RSS but all the other categories showed comparable, lower levels. The same was observed for BRSC scores (Supplementary Figure S8d–f). These results suggest the inherent biology uncovered might be specific to pCR rather than good response to radiotherapy.

### Biological assessment of RSS

**Fig. 4:**
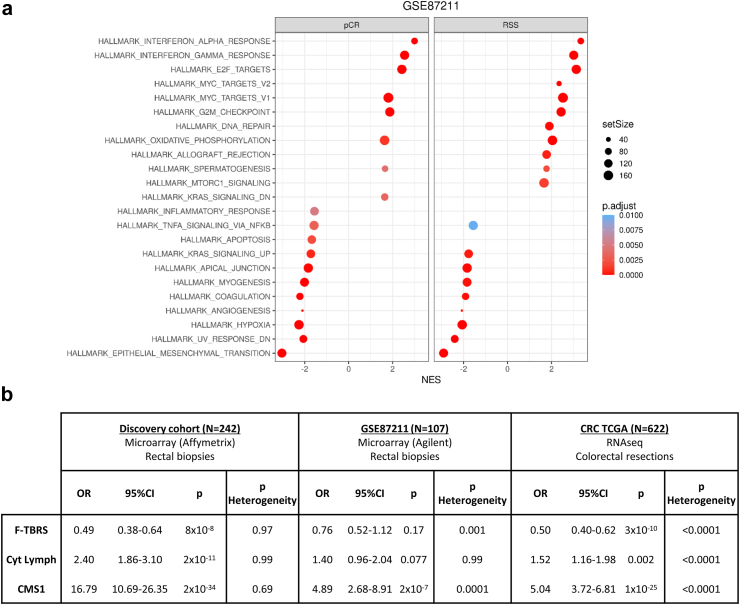
**RSS biology** a. GSEA for cancer hallmarks from differential expression analyses based on pCR and RSS prediction in GSE87211. b. Association of BRSC molecular features and RSS genes by meta-analysis for all genes in discovery cohort, GSE87211 and TCGA separately (see Supplementary Figure S8 for full analysis).

In order to understand the biology associated with RSS, we first performed gene set enrichment analysis (GSEA) of the RSS model predictions on GSE87211 using hallmark genesets. We compared “RSS positive” (eg predicted to be pCRs) against “RSS negative” (eg predicted to be not-pCRs). The same analysis was also performed with real pCR status for a fair comparison (Fig. 4a). We identified 15 hallmarks in common between both analyses, all of them in the same direction, while four hallmarks were identified only by RSS predicted status and also four only by pCR status. These results suggest strong concordance of RSS prediction with actual pCR status also at the level of biological pathways. In agreement with our previous BRSC results, two of the shared hallmarks showing stronger enrichment in pCRs were immune-related (interferon alpha response and interferon gamma response) while a hallmark tightly associated with stroma (epithelial mesenchymal transition) was strongly enriched in not-pCRs cases. Some other interesting hallmarks linked in the literature with radiotherapy were found such as hypoxia and oxidative phosphorylation.

We then aimed at comparing RSS with our BRSC model that underlines the biology driving pCR. The association of each individual RSS gene with each BRSC biological variable was analysed by regression and meta-analysed. As expected, the discovery set showed strong association for all 3 BRSC features without heterogeneity in the models. GSE87211 showed the expected trends although in this smaller cohort with more heterogeneity, two of the variables did not reach significance. The same analysis was then performed on RNAseq data from TCGA CRC resections. In this third large set all 3 BRSC features were significantly associated with RSS genes, albeit with high levels of heterogeneity (Fig. 4b and Supplementary Figure S9). These analyses suggest the 33 RSS genes broadly overlap with all 3 BRSC features.

Our biological analyses suggest RSS is strongly associated with the tumour microenvironment. Accordingly, we aimed at testing whether the expression of these 33 genes may potentially be driven by the composition of different cell types in the profiled tissue. Using an unrelated transcriptome cohort composed of four different cell types separated by FACS from six CRC cases,^18^ we identified 14 RSS genes strongly expressed in specific cell types (epithelial, leucocytes, endothelial or fibroblasts) and 12 genes that were not (Supplementary Figure S10).

### Comparison with similar published signatures

**Fig. 5:**
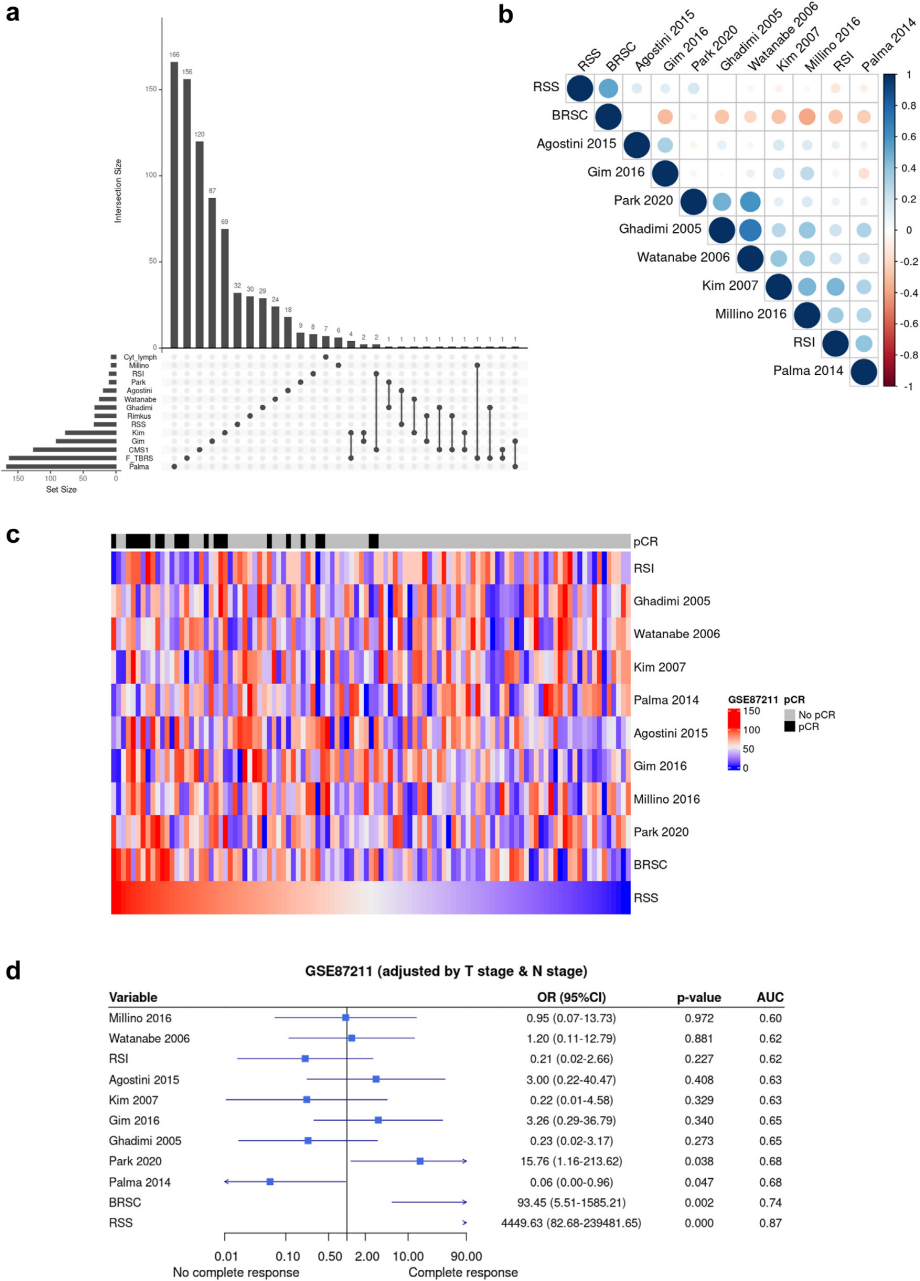
**Comparison of our signatures with published ones**. a. Number of overlapping entrez genes in published signatures and ours. b. Pearson correlation across different signatures in GSE87211. c. Heatmap of samples ranked by each signature sorted by biological score of RSS in GSE87211. d. Prediction to pCR in GSE87211 by each signature sorted by AUC.

We compared RSS and BRSC with similar signatures reported in the literature to predict outcome to radiotherapy in RC (Supplementary Table S1). We first found very few overlapping genes between any of the signatures (Fig. 5a, Supplementary Figure S11a). We then looked for correlations in GSE87211 which was not used for training any of the signatures. RSS was only correlated with BRSC but not with any of the published signatures (Fig. 5b). We then compared how samples would have been ranked according to each signature. None of them seem to show similar ranks to RSS (Fig. 5c) but most of them seemed to show an inverse association for BRSC (Supplementary Figure S11b). Finally, we tested the prediction ability for pCR using each signature score. Only Palma and Park were statistically significant but at much lower level than BRSC and RSS (p = 0.047, p = 0.038, p = 0.002 and p < 0.001 respectively). Notably, better performance of our two models was clearly evidenced by better ORs and AUCs (Fig. 5d). In summary, most of these published signatures mildly associate with the same biology we have identified but they all clearly underperfom to predict pCR compared to RSS.

## Discussion

CRC biology remains a challenge to the research and clinical communities. The 15% of MSI cases shows the importance of the interaction between epithelial cell biology and immune response as key determinant of outcome. The development of the CMS classification has helped to stratify the remaining 85% of CRC with proficient mismatch repair.^19^ While MSI cases are allocated with some other cases with immune activation to CMS1, the key observation from the CMS and prior gene expression classification systems has been the identification of CMS4 with its increase in fibrotic stroma and prevalence of TGFβ signalling, poor prognosis and poor response to chemotherapy. Recently, we reported that image based consensus molecular subtypes correlate with radiation response with increased pCRs in imCMS1, but reduced pCRs in imCMS4.^20^

This study has shown that the predictors associated with pCR to radiotherapy are based on contributions from the tumour microenvironment, notably stromal cells (TGFβ signalling) and immune response (CMS1, cytotoxic lymphocytes). Increasing evidence points to the immune response as being a critical determinant of radiation sensitivity. Using 97 rectal biopsies from patients treated with heterogeneous neoadjuvant radiotherapy regimens, estimations of immune infiltrate were found associated with increased response and prolonged disease free survival.^21^ In a separate report, gene expression from over 10,000 pancancer patients showed high RSI^22^ had distinct enrichment of interferon-associated signalling pathways and immune cell infiltrates.^23^ The importance of an IL-1 mediated response from inflammatory cancer associated fibroblasts has been shown to mediate radiation resistance in RC.^24^ Consistently, here we report radiation response associates with TGFβ and immune markers. TGFβ is a pleiotropic cytokine derived from many cell types but especially the myofibroblastic stromal cells, which is present at a high level in stroma-rich CRC,^19^ is released from its extracellular matrix trap by radiotherapy,^20^ reduces intrinsic radiosensitivity through DNA repair pathway switching^25^ and has multiple inhibitory effects on the immune response.^26^ These predictive features are plausible biologically and targetable, enabling design of combination therapy through inhibition of TGFβ or related stromal cytokine pathways, such as IL-1, and enhancing the immune cell engagement through immune checkpoint inhibition and other related approaches. Interestingly, this biology overlaps extensively with that driving the development of metastasis in the poor prognosis CMS4 subtype. Batlle and colleagues have shown in an autochthonous tumour model of CMS4 (APTK) which is highly metastatic, inhibition of TGFβ and with PD-1 inhibition is the most potent approach to inhibiting the development of metastases and enhancing survival.^27^ This approach has been shown in early clinical trials using the TGFBR1 inhibitor vactosertib in metastatic CRC in combination with pembrolizumab.^28^

We complemented this hypothesis-based approach with a hypothesis-free, ML method undertaken separately using the same datasets. The availability of more complex and personalised statistical tools for gene expression data analysis coupled with an unprecedented large dataset enabled us to derive robust biomarkers. A dataset without proper pre-processing to handle batch effect, causal inference, class imbalance and outliers can lead to misleading results.^29^ Given the validation set was processed in an entirely different laboratory setting, we performed batch correction. This requires disclosure of clinical variables, including response, in order to avoid degrading the biological signal. However, we also performed an analysis not revealing the response data during batch correction, which is suboptimal from a batch correction perspective, but avoids disclosing the response data. We also used a set of pre-processing techniques including correction of class imbalance using downsampling to balance classes in the discovery cohort and multiple iterations using differing not-pCR cases to minimise biases in our discovery cohort. After addressing most of the known biases, DEG analysis was done on 100 subsets of the discovery cohort with two tools finding a robust geneset of 80 DEGs in all iterations. Finally, we used six well known ML algorithms trained on all 80 DEGs or selected features to make prediction models and internal validation was undertaken using 10-fold cross-validation. This identified a RSS of 33 genes which is independent of T and N stage and highly predictive of pCR with high robustness and accuracy in an independent dataset with optimal pre-processing, which remained significant when omitting pCR from the combat step. GSEA analysis of samples with either high RSS scores or with actual pCR, showed interferon alpha response and interferon gamma response were highly enriched in responding patients while a hallmark tightly associated with stroma (epithelial mesenchymal transition) was strongly enriched in not-pCRs cases showing the two methods had independently shown the same biological features.

**Fig. 6:**
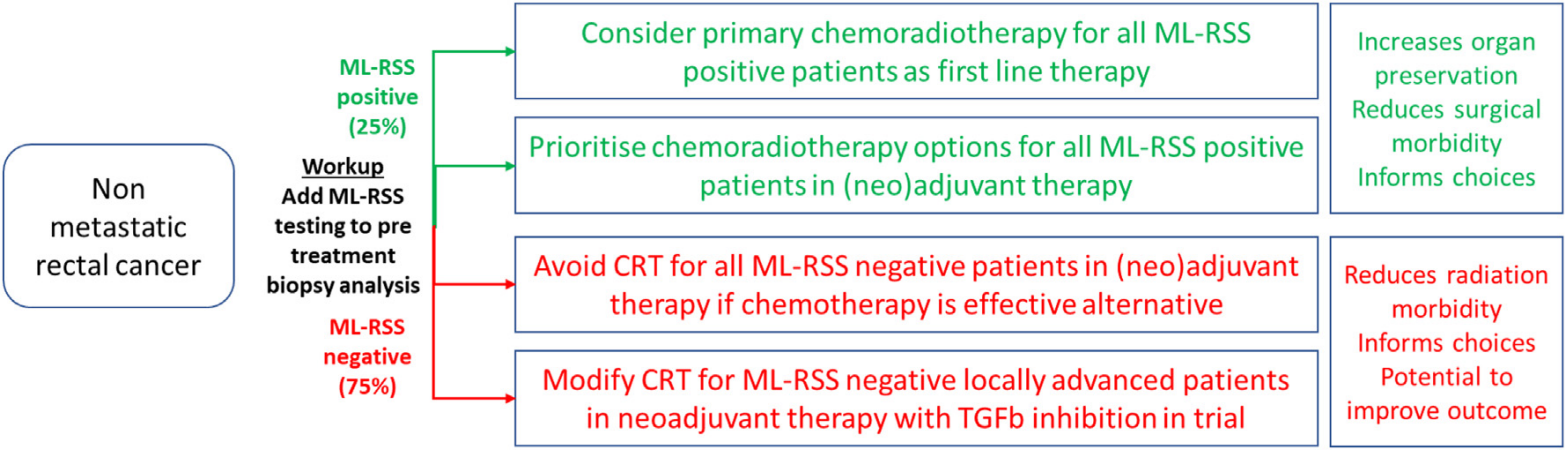
**Clinical implementation of RSS**. NCCN guidelines define clinical options for patients with rectal cancer. Adding a certified test based on the 33 gene signature described here would inform decision making, resulting in increased organ preservation, reduced treatment morbidity, and has potential to improve outcomes.

The ML approach based on rigorous statistics has therefore provided us with the basis for a biomarker which could be tested prospectively to identify patients with RC who are highly likely to achieve pCR to standard dose radiotherapy coupled with fluoropyrimidines. The 15–25% of patients who have the radiosensitive biology could be identified at diagnosis and counselled that a primary radiotherapy based approach to management is possible similar to that currently undertaken in anal cancer, with planned close follow up on a ‘watch and wait’ basis as is currently performed in those found to have a complete clinical response after neoadjuvant CRT. Conversely, it identifies patients unlikely to obtain pCR who may be considered for trials targeting the stromal inhibitory mechanisms (TGFβ) and enhancing the immune response in combination with radiation. Further research may also test whether RSS may also be useful in earlier stage disease for patients currently not considered for CRT who may achieve an organ preserving strategy (Fig. 6, Supplementary Figure S12).

Only recently RNA profiling has been successfully undertaken using optimised 3’ RNAseq from FFPE tissue. In our study we used the Almac Xcel array which is optimised for FFPE samples and we were able to achieve good yields from 98% of rectal biopsies. In collaboration with the Wellcome Trust Sanger Institute we also developed a reduced input to the sequencing process enabling 50 ng DNA to be used rather than the previous minimum standard of 200 ng. The use of FFPE obviously makes collection of cases much more straightforward and also makes the results more readily implementable in the clinical workflow as fresh frozen samples are not required.

The main strengths of our study compared to previous efforts are larger discovery and validation sets, strong sample selection resulting in clear clinical homogeneity, adjustment of the potential confounders T and N baseline stage, assessment of the associated biology, additional use of mutation and copy number data, and the use of a clear endpoint both clinically and biologically such as pCR. Although the latter may have resulted in some loss of signal from good responders not reaching pCR, our biomarkers showed similar low levels across responses and yT stage within non-pCRs. This may be expected since they were derived specifically for pCR. Overall, our results provide a comprehensive picture that was missing in this field.

Our research is limited to analysis of patients treated with long course CRT. Total noadjuvant chemotherapy (TNT) is an emerging standard of care based on randomised trials^30^ and meta-analyses^31^^,^^32^ showing improved outcomes. Recently, we have assessed predictors of response with varying chemoradiotherapy regimens and show that the addition of oxaliplatin to CRT seems to reduce the negative predictive effect of stromal biology on pCR.^33^ Further research, including acquisition of transcriptomics data in the relevant cohorts, will be needed to fully determine whether our findings may apply to patients treated with TNT, and also to stage I and IV patients as they were mostly missing in our cohorts. It is also unclear whether the same level of signal may be shown in patients with regimens other than with addition of fluoropyrimidines or even in different tumour types. RSS needs further development using a standardised analytical laboratory process to avoid the requirement for batch correction and to be further validated in independent datasets using that locked down protocol to become a clinically validated tool. To our knowledge this is the first time that a predictive model has been used to predict individual patient outcome in an entirely independent dataset. Finally, while our two biomarkers do not show heterogeneity in our three curated cohorts, we also show early evidence that the signals from some of the individual RSS genes may come from different cell lineages. More studies are needed to properly detail the involvement of each RSS gene in different cell types and why their combination results in stable signals to predict pCR in rectal biopsies. Such analyses may also consider that while we identify three biological features independently associated with pCR in multivariable analysis (F-TBRS, CL, CMS1), six others were also found in univariable models and it can not be ruled out that any of them or others not profiled in this study may have a relevant role.

In summary, RCs that respond completely to radiotherapy have the biological characteristics of immune activation as identified by CMS1 and cytotoxic lymphocytic infiltration and an absence of immune inhibitory TGFβ signalling. These tumours may be identified by measurement of expression of a 33 geneset that merits further research for validation and development as a potential new biomarker. This could open the door to selection of responsive patients for treatment of patients with RC with a primary radiotherapy based approach as for anal cancer. Conversely those with predicted radioresistance would be candidates for clinical trials evaluating addition of immune-oncology agents and stromal signalling inhibition to overcome these drivers of immune exclusion and radioresistance.

## Contributors

This is a publication by the S:CORT consortium. Enric Domingo undertook the hypothesis based analysis, Sanjay Rathee undertook the machine learning analysis, Andrew Blake collated and curated the clinical and molecular data on behalf of the S:CORT consortium. Les Samuel and Graeme Murray provided the sample set and associated anonymised clinical data from Aberdeen (Grampian cohort). David Sebag-Montefiore, Simon Gollins, Nicholas West and Rubina Begum led the Aristotle trial and provided the sample set and data from the Aristotle control arm. Alessandro Barberis, Sylvana Hassanieh, Umair Mahmood contributed to the bioinformatic analysis of the data which was planned and overseen by Francesca Buffa. Susan Richman and Phil Quirke undertook the histological quality assessment, sectioning, DNA extraction of all samples in the University of Leeds. Viktor Koelzer undertook a second pathologist check of all histology sections. Keara Redmond and Philip Dunne undertook the RNA expression analysis in Queens University Belfast. Aikaterini Chatzipli and Ultan McDermott designed and undertook the DNA sequencing and associated bioinformatics at the Wellcome Trust Sanger Institute. Francesca Buffa conceived the development of the machine learning classifier, designed the ML study and supervised the analyses. Simon Leedham, Ian Tomlinson and Tim Maughan conceived the project, obtained the funding and oversaw the project.

The paper was drafted by Tim Maughan, Enric Domingo, Francesca Buffa and Sanjay Rathee. All authors have reviewed and approved the final manuscript. All authors had access to the data and ED, SR and AB directly accessed and verified the raw data before final submission.

The S:CORT consortium membership contributed to the overall delivery of the programme including idea generation, sample acquisition, molecular analyses and statistical analyses.

## Data sharing statement

The transcriptomic data from both S:CORT cohorts used in this study is publicly available in the following links: https://www.s-cort.org/sites/default/files/exports/scort_ws3_grampian_export_84m9fndk/ws3_grampian_expression_raw.zip and https://www.s-cort.org/sites/default/files/exports/scort_ws3_aristotle_export_6ythgf78/ws3_aristotle_expression_raw.zip. The list of S:CORT sample ids specifically used in this study are in https://www.s-cort.org/sites/default/files/exports/sample_sheets/SCORT_samples_Domingo_EBioMedicine.csv.

Additional S:CORT data is available to all academic researchers on submission of a data request to the data access committee. For commercial agencies, the data will be made available through Cancer Research Horizons acting on behalf of the funders and consortium members. An implementation of these models with hyperparameters is available on GitHub (https://github.com/sanjaysinghrathi/SCORT-ML-Pipeline).

## Declaration of interests

TSM is now employed by the University of Liverpool and acknowledges consultancy payments from Astrazeneca, Ground Truth Laboratories and Nordic Pharma. V.H.K. has served as an invited speaker on behalf of Indica Labs. U.M is now employed by and holds stocks in Astrazeneca. Other authors declare no conflict of interests.

## Appendix A Supplementary data

Supplementary Tables

Supplementary Figures Figure 1. Consort diagrams for sample selection in Grampian (a), Aristotle (b) and GSE87211 (c) cohorts. Figure 2. a. Frequencies of binary variables and mean(IQR) of continuous variables in Aristotle and Grampian (discovery cohort). b. Histograms of continuous variables in discovery cohort (Aristotle and Grampian combined). Figure 3. Pre-processing of transcriptomic and clinical data. a. PCA (Principal Component Analysis) plot to show batch effect in Grampian and Aristotle cohort. b.PCA plot to show Grampian and Aristotle cohort after batch correction using ComBat. c. Distribution of tumour and nodal stages in discovery cohort. d. Binarized tumour and nodal stages in discovery cohort. e. Effect of class imbalance on separation line. f. Discovery cohort after downsampling to correct for class imbalance. Figure 4. Meta-analysis of the three significant predictors of pCR in the three cohorts shows no significant heterogeneity. Figure 5. Exploratory analyses of variables from copy number (a) and mutations (b) in the discovery cohort. Figure 6. Summary of ML pipeline methods. a. Volcano plot for global limma results. b. Algorithm 1 showing the whole process step-by-step. c. Histogram of p-values. d. Significant genes for 100 iteration. e. Decision making genes using Boruta Figure 7. Alternative RSS models. a. PCA to show technical batch effect in discovery and validation cohort. b-d. ROC curve, score plot and confusion matrix for Gradient boosting machine model on a balanced subset of GSE87211. e-g. ROC curve, score plot and confusion matrix for GBM model on balanced GSE87211 agnostic for pCR. h. AUC on discovery cohort for random 1000 gene sets. i. AUC on balanced validation cohort for random 1000 gene sets. j. AUC on balanced validation cohort prognostic of pCR for random 1000 gene sets. Figure 8. RSS and BRSC are similar across samples without pCR. RSS scores are shown as boxplots split by response in Grampian (a), yT stage in discovery cohort (b) and yT stage in GSE87211 (c). BRSC scores are are shown as boxplots split by response in Grampian (d), yT stage in discovery cohort (e) and yT stage in GSE87211 (f). Figure 9. Associations of the 3 BRSC molecular features with each of the RSS genes in 3 different cohorts (Discovery cohort, GSE87211 and CRC TCGA) and metaanalysis.∗genes where expression has been flipped for this analysis as they are associated with lack of pCR. Figure 10. Expression heatmap of RSS genes in 4 different cell types from GSE39396. Figure 11. a. Number of overlapping entrez genes in published signatures, our new signatures and all the ones used in the candidate analysis. b. Heatmap of samples ranked by each signature sorted by biological score of BRSC in GSE87211. Figure 12. Clinical implementation of RSS. Potential alterations to NCCN guidelines for rectal cancer management if a certified test using the ML-RSS was available, based on data from the validation set.

## References

[1] Chemotherapy, radiotherapy and tumour resections in England: 2013-2021. https://digital.nhs.uk/data-and-information/publications/statistical/chemotherapy-radiotherapy-and-surgical-tumour-resections-in-england/2013-to-2021.

[2] Benson A.B., Venook A.P., Al-Hawary M.M. (2020). NCCN guidelines insights: rectal cancer, version 6.2020. J Natl Compr Canc Netw.

[3] Yu G., Lu W., Jiao Z., Qiao J., Ma S., Liu X. (2021). A meta-analysis of the watch-and-wait strategy versus total mesorectal excision for rectal cancer exhibiting complete clinical response after neoadjuvant chemoradiotherapy. World J Surg Oncol.

[4] Ghadimi B.M., Grade M., Difilippantonio M.J. (2005). Effectiveness of gene expression profiling for response prediction of rectal adenocarcinomas to preoperative chemoradiotherapy. J Clin Oncol.

[5] Gim J., Cho Y.B., Hong H.K. (2016). Predicting multi-class responses to preoperative chemoradiotherapy in rectal cancer patients. Radiat Oncol.

[6] Kim I.J., Lim S.B., Kang H.C. (2007). Microarray gene expression profiling for predicting complete response to preoperative chemoradiotherapy in patients with advanced rectal cancer. Dis Colon Rectum.

[7] Park I.J., Yu Y.S., Mustafa B. (2020). A nine-gene signature for predicting the response to preoperative chemoradiotherapy in patients with locally advanced rectal cancer. Cancers (Basel).

[8] Millino C., Maretto I., Pacchioni B. (2017). Gene and MicroRNA expression are predictive of tumor response in rectal adenocarcinoma patients treated with preoperative chemoradiotherapy. J Cell Physiol.

[9] Watanabe T., Komuro Y., Kiyomatsu T. (2006). Prediction of sensitivity of rectal cancer cells in response to preoperative radiotherapy by DNA microarray analysis of gene expression profiles. Cancer Res.

[10] Rimkus C., Friederichs J., Boulesteix A.L. (2008). Microarray-based prediction of tumor response to neoadjuvant radiochemotherapy of patients with locally advanced rectal cancer. Clin Gastroenterol Hepatol.

[11] Palma P., Cano C., Conde-Muiño R. (2014). Expression profiling of rectal tumors defines response to neoadjuvant treatment related genes. PLoS One.

[12] Agostini M., Zangrando A., Pastrello C. (2015). A functional biological network centered on XRCC3: a new possible marker of chemoradiotherapy resistance in rectal cancer patients. Cancer Biol Ther.

[13] Fokas E., Fietkau R., Hartmann A. (2018). Neoadjuvant rectal score as individual-level surrogate for disease-free survival in rectal cancer in the CAO/ARO/AIO-04 randomized phase III trial. Ann Oncol.

[14] Hu Y., Gaedcke J., Emons G. (2018). Colorectal cancer susceptibility loci as predictive markers of rectal cancer prognosis after surgery. Genes Chromosomes Cancer.

[15] Burrell R.A., McClelland S.E., Endesfelder D. (2013). Replication stress links structural and numerical cancer chromosomal instability. Nature.

[16] Barberis A., Aerts H.J.W.L., Buffa F.M. (2024). Robustness and reproducibility for AI learning in biomedical sciences: RENOIR. Sci Rep.

[17] Proposed regulatory framework for modifications to artificial intelligence/machine learning (AI/ML)-based software as a medical device (SaMD). https://www.fda.gov/files/medical%20devices/published/US-FDA-Artificial-Intelligence-and-Machine-Learning-Discussion-Paper.pdf.

[18] Fisher N.C., Byrne R.M., Leslie H. (2022). Biological misinterpretation of transcriptional signatures in tumor samples can unknowingly undermine mechanistic understanding and faithful alignment with preclinical data. Clin Cancer Res.

[19] Guinney J., Dienstmann R., Wang X. (2015). The consensus molecular subtypes of colorectal cancer. Nat Med.

[20] Lafarge M.W., Domingo E., Sirinukunwattana K. (2024). Image-based consensus molecular subtyping in rectal cancer biopsies and response to neoadjuvant chemoradiotherapy.. NPJ Precis Oncol.

[21] Chatila W.K., Kim J.K., Walch H. (2022). Genomic and transcriptomic determinants of response to neoadjuvant therapy in rectal cancer. Nat Med.

[22] Chatila W.K., Kim J.K., Walch H. (2009). Systems biology modeling of the radiation sensitivity network: a biomarker discovery platform. Int J Radiat Oncol Biol Phys.

[23] Grass G.D., Alfonso J.C.L., Welsh E. (2022). The radiosensitivity index gene signature identifies distinct tumor immune microenvironment characteristics associated with susceptibility to radiation therapy. Int J Radiat Oncol Biol Phys.

[24] Nicolas A.M., Pesic M., Engel E. (2022). Inflammatory fibroblasts mediate resistance to neoadjuvant therapy in rectal cancer. Cancer Cell.

[25] Liu Q., Lopez K., Murnane J., Humphrey T., Barcellos-Hoff M.H. (2019). Misrepair in context: TGFβ regulation of DNA repair. Front Oncol.

[26] Batlle E., Massague J. (2019). Transforming growth factor-beta signaling in immunity and cancer. Immunity.

[27] Tauriello D.V.F., Palomo-Ponce S., Stork D. (2018). TGFbeta drives immune evasion in genetically reconstituted colon cancer metastasis. Nature.

[28] Kim T.W., Lee K.W., Ahn J.B., Hong Y.S. (2021). Efficacy and safety of vactosertib and pembrolizumab combination in patients with previously treated microsatellite stable metastatic colorectal cancer. J Clin Oncol.

[29] Zhang Y., Parmigiani G., Johnson W.E. (2020). ComBat-seq: batch effect adjustment for RNA-seq count data. NAR Genom Bioinform.

[30] Bahadoer R.R., Dijkstra E.A., van Etten B. (2021). Short-course radiotherapy followed by chemotherapy before total mesorectal excision (TME) versus preoperative chemoradiotherapy, TME, and optional adjuvant chemotherapy in locally advanced rectal cancer (RAPIDO): a randomised, open-label, phase 3 trial. Lancet Oncol.

[31] Liu S., Jiang T., Xiao L. (2021). Total neoadjuvant therapy (TNT) versus standard neoadjuvant chemoradiotherapy for locally advanced rectal cancer: a systematic review and meta-analysis. Oncologist.

[32] Kasi A., Abbasi S., Handa S. (2020). Total neoadjuvant therapy vs standard therapy in locally advanced rectal cancer: a systematic review and meta-analysis. JAMA Netw Open.

[33] Mahmood U., Blake A., Rathee S (2024). Stratification to neoadjuvant radiotherapy in rectal cancer by regimen and transcriptional signatures. Cancer Research Comms.

