# Supplementary material for: Identification and validation of a machine learning model of complete response to radiation in rectal cancer reveals immune infiltrate and TGFβ as key predictors
Source: eBioMedicine. 2024 Jul 16;106:105228. doi: 10.1016/j.ebiom.2024.105228 (PMC11663784; doi:10.1016/j.ebiom.2024.105228)
Supplement: Supplementary Tables [file mmc1.docx]

**Supp Table 1.** Summary of papers reporting stratifiers for response to neoadjuvant radiotherapy in rectal cancer.

**Supp Table 2.** Summary of predefined candidates for response to neoadjuvant radiotherapy in rectal cancer.

**Supp Table 3.** Summary of clinical and molecular profiles by cohort.

| **Variable** | **N** | **Grampian,**  **N = 125^1^** | **Aristotle,**  **N = 124^1^** | **GSE87211,**  **N = 107^1^** | **p-value^2^** |
| --- | --- | --- | --- | --- | --- |
| **pCR** | 356 | 33 (26%) | 24 (19%) | 22 (21%) | 0.4 |
| **RT dose (Gy)** | 356 | 45-50.4 | 45-50.4 | 50.4 | - |
| **RT fractions** | 356 | 25 | 25 | 28 | - |
| **Chemotherapy** | 356 |  |  |  | - |
| Capecitabine |  | 125 (100%) | 124 (100%) | 0 |  |
| Infusional 5FU |  | 0 | 0 | 107 (100%) |  |
| **pT** | 356 |  |  |  | <0.001 |
| 1 |  | 1 (0.8%) | 0 (0%) | 0 (0%) |  |
| 2 |  | 19 (15%) | 6 (4.8%) | 4 (3.7%) |  |
| 3 |  | 98 (78%) | 96 (77%) | 97 (91%) |  |
| 4 |  | 7 (5.6%) | 22 (18%) | 6 (5.6%) |  |
| **pN** | 356 |  |  |  | <0.001 |
| 0 |  | 61 (49%) | 27 (22%) | 39 (36%) |  |
| 1 |  | 51 (41%) | 58 (47%) | 68 (64%) |  |
| 2 |  | 13 (10%) | 39 (31%) | 0 (0%) |  |
| **CMS** | 349 |  |  |  | 0.5 |
| CMS1 |  | 12 (9.6%) | 11 (8.9%) | 9 (8.4%) |  |
| CMS2 |  | 19 (15%) | 16 (13%) | 19 (18%) |  |
| CMS3 |  | 17 (14%) | 18 (15%) | 25 (23%) |  |
| CMS4 |  | 45 (36%) | 46 (37%) | 35 (33%) |  |
| UNK |  | 32 (26%) | 33 (27%) | 19 (18%) |  |
| **APC mut** | 224 | 94 (90%) | 94 (79%) | NR | 0.032 |
| **TP53 mut** | 224 | 87 (83%) | 85 (71%) | NR | 0.043 |
| **KRAS mut** | 224 | 60 (57%) | 69 (58%) | NR | 0.9 |
| **CIN+** | 224 | 80 (76%) | 74 (62%) | NR | 0.024 |
| **Hypermutation (MSI/POLE)** | 224 | 3 (2.9%) | 1 (0.8%) | NR | 0.3 |
| ^1^n (%) | | | | | |
| ^2^Pearson's Chi-squared test; Fisher's exact test | | | | | |
| Gy: Grays; NR: Not Reported; MSI: MicroSatellite Instability | | | | | |

**Supp Table 4.** RSS signature (33 probesets and annotated genes).

| **Probe.Set.ID** | **Gene.Symbol** | **Entrez.Gene** | **Direction association** |
| --- | --- | --- | --- |
| ADXEC.3431.C1_at | ADH5 | 128 | Lack of pCR (negative) |
| ADXEC.16192.C1_at | ALOX5AP | 241 | Lack of pCR (negative) |
| ADXEC.17982.C1_at | ATF7 | 11016 | Lack of pCR (negative) |
| ADXEC.28557.C1_at | C7orf50 | 84310 | pCR (positive) |
| ADXECRS.26433_at | CACTIN-AS1 | 404665 | pCR (positive) |
| ADXEC.17263.C1-a_s_at | CITED2 | 10370 | Lack of pCR (negative) |
| ADXEC.2350.C1_s_at | CYBA | 1535 | pCR (positive) |
| ADXEC.7366.C1_at | EXOC1 | 55763 | Lack of pCR (negative) |
| ADXECEMUTR.2871_x_at | FTH1P3 | 2498 | pCR (positive) |
| ADXEC.4765.C1_s_at | GLG1 | 2734 | Lack of pCR (negative) |
| ADXEC.19644.C1_s_at | HNRNPA0 | 10949 | Lack of pCR (negative) |
| ADXEC.20850.C1_at | IL12RB1 | 3594 | pCR (positive) |
| ADXEC.33316.C1_at | KLK14 | 43847 | pCR (positive) |
| ADXEC.17.C2_s_at | MAGED2 | 10916 | Lack of pCR (negative) |
| ADXEC.17669.C1_at | MAST4 | 375449 | Lack of pCR (negative) |
| ADXEC.3309.C1_at | MICU2 | 221154 | Lack of pCR (negative) |
| ADXEC.3241.C1_at | MTG1 | 92170 | pCR (positive) |
| ADXEC.15626.C2_s_at | NACC1 | 112939 | pCR (positive) |
| ADXEC.12129.C1_at | PHF20L1 | 51105 | Lack of pCR (negative) |
| ADXEC.3766.C1_s_at | PPCDC | 60490 | pCR (positive) |
| ADXECRS.18612_at | PXT1 | 222659 | pCR (positive) |
| ADXEC.12091.C1_s_at | RNASE4 | 6038 | Lack of pCR (negative) |
| ADXEC.15941.C1_at | RND1 | 27289 | pCR (positive) |
| ADXEC.431.C1-a_s_at | RSL24D1 | 51187 | Lack of pCR (negative) |
| ADXECRS.17670_at | SNORD74 | 619498 | pCR (positive) |
| ADXEC.5281.C1_at | SPR | 6697 | pCR (positive) |
| ADXECNTDJ.1561_s_at | SURF2 | 6835 | pCR (positive) |
| ADXEC.1200.C1_at | TCEAL4 | 79921 | Lack of pCR (negative) |
| ADXEC.3214.C1_s_at | TMEM176A | 55365 | Lack of pCR (negative) |
| ADXECRS.32553_at | TUBA8 | 51807 | pCR (positive) |
| ADXEC.22722.C1_s_at | USP30-AS1 | 100131733 | pCR (positive) |
| ADXECRS.11683_s_at | YBX1P4 | 100131012 | pCR (positive) |
| ADXEC.26562.C1_at | ZNF585B | 92285 | Lack of pCR (negative) |

**Supp Table 5.** Checklist of TRIPOD guidelines for Prediction Model and Validation.

| **Section/Topic** | **Item** |  | **Checklist Item** | **Page** |
| --- | --- | --- | --- | --- |
| **Title and abstract** | | | | |
| Title | 1 | D;V | Identify the study as developing and/or validating a multivariable prediction model, the target population, and the outcome to be predicted. | p1 |
| Abstract | 2 | D;V | Provide a summary of objectives, study design, setting, participants, sample size, predictors, outcome, statistical analysis, results, and conclusions. | p4 |
| **Introduction** | | | | |
| Background and objectives | 3a | D;V | Explain the medical context (including whether diagnostic or prognostic) and rationale for developing or validating the multivariable prediction model, including references to existing models. | p6-7 |
|  | 3b | D;V | Specify the objectives, including whether the study describes the development or validation of the model or both. | p6-7 |
| **Methods** | | | | |
| Source of data | 4a | D;V | Describe the study design or source of data (e.g., randomized trial, cohort, or registry data), separately for the development and validation data sets, if applicable. | p7 |
|  | 4b | D;V | Specify the key study dates, including start of accrual; end of accrual; and, if applicable, end of follow-up. | Supp Meth p1 |
| Participants | 5a | D;V | Specify key elements of the study setting (e.g., primary care, secondary care, general population) including number and location of centres. | p7, Supp Meth p1 |
|  | 5b | D;V | Describe eligibility criteria for participants. | p7, Supp Meth p1 |
|  | 5c | D;V | Give details of treatments received, if relevant. | p7 |
| Outcome | 6a | D;V | Clearly define the outcome that is predicted by the prediction model, including how and when assessed. | p7 |
|  | 6b | D;V | Report any actions to blind assessment of the outcome to be predicted. | Supp Meth p1-2 |
| Predictors | 7a | D;V | Clearly define all predictors used in developing or validating the multivariable prediction model, including how and when they were measured. | p8, Supp Meth p4-11 |
|  | 7b | D;V | Report any actions to blind assessment of predictors for the outcome and other predictors. | Supp Meth p1,2,12 |
| Sample size | 8 | D;V | Explain how the study size was arrived at. | p7, Supp Meth p1 |
| Missing data | 9 | D;V | Describe how missing data were handled (e.g., complete-case analysis, single imputation, multiple imputation) with details of any imputation method. | p1 |
| Statistical analysis methods | 10a | D | Describe how predictors were handled in the analyses. | Supp Meth p4-11 |
|  | 10b | D | Specify type of model, all model-building procedures (including any predictor selection), and method for internal validation. | Supp Meth p4-11 |
|  | 10c | V | For validation, describe how the predictions were calculated. | Supp Meth p9-11 |
|  | 10d | D;V | Specify all measures used to assess model performance and, if relevant, to compare multiple models. | Supp Meth p9-11 |
|  | 10e | V | Describe any model updating (e.g., recalibration) arising from the validation, if done. | NA |
| Risk groups | 11 | D;V | Provide details on how risk groups were created, if done. | Supp Meth p9-11 |
| Development vs. validation | 12 | V | For validation, identify any differences from the development data in setting, eligibility criteria, outcome, and predictors. | p7, Supp Meth p2 |
| **Results** | | | | |
| Participants | 13a | D;V | Describe the flow of participants through the study, including the number of participants with and without the outcome and, if applicable, a summary of the follow-up time. A diagram may be helpful. | Fig 1, Supp Fig 1 |
|  | 13b | D;V | Describe the characteristics of the participants (basic demographics, clinical features, available predictors), including the number of participants with missing data for predictors and outcome. | P7-9, Supp Meth p1-2, Fig 1 |
|  | 13c | V | For validation, show a comparison with the development data of the distribution of important variables (demographics, predictors and outcome). | Fig 2,3,5 |
| Model development | 14a | D | Specify the number of participants and outcome events in each analysis. | P7, Supp Meth p1-2, Fig 1 |
|  | 14b | D | If done, report the unadjusted association between each candidate predictor and outcome. | Fig 2, Supp Fig 9 |
| Model specification | 15a | D | Present the full prediction model to allow predictions for individuals (i.e., all regression coefficients, and model intercept or baseline survival at a given time point). | Fig 2, 3 |
|  | 15b | D | Explain how to the use the prediction model. | Supp Meth 9-11 |
| Model performance | 16 | D;V | Report performance measures (with CIs) for the prediction model. | Fig 2, 5 |
| Model-updating | 17 | V | If done, report the results from any model updating (i.e., model specification, model performance). | NA |
| **Discussion** | | | | |
| Limitations | 18 | D;V | Discuss any limitations of the study (such as nonrepresentative sample, few events per predictor, missing data). | p12-16 |
| Interpretation | 19a | V | For validation, discuss the results with reference to performance in the development data, and any other validation data. | p12-16 |
|  | 19b | D;V | Give an overall interpretation of the results, considering objectives, limitations, results from similar studies, and other relevant evidence. | p12-16 |
| Implications | 20 | D;V | Discuss the potential clinical use of the model and implications for future research. | p12-16 |
| **Other information** | | | | |
| Supplementary information | 21 | D;V | Provide information about the availability of supplementary resources, such as study protocol, Web calculator, and data sets. | p3 |
| Funding | 22 | D;V | Give the source of funding and the role of the funders for the present study. | p3 |
