# Supplementary material for: Identification and validation of a machine learning model of complete response to radiation in rectal cancer reveals immune infiltrate and TGFβ as key predictors
Source: eBioMedicine. 2024 Jul 16;106:105228. doi: 10.1016/j.ebiom.2024.105228 (PMC11663784; doi:10.1016/j.ebiom.2024.105228)
Supplement: Supplementary Figures — Figure 1. Consort diagrams for sample selection in Grampian (a), Aristotle (b) and GSE87211 (c) cohorts. Figure 2. a. Frequencies of binary variables and mean(IQR) of continuous variables in Aristotle and Grampian (discovery cohort). b. Histograms of continuous variables in discovery cohort (Aristotle and Grampian combined). Figure 3. Pre-processing of transcriptomic and clinical data. a. PCA (Principal Component Analysis) plot to show batch effect in Grampian and Aristotle cohort. b.PCA plot to show Grampian and Aristotle cohort after batch correction using ComBat. c. Distribution of tumour and nodal stages in discovery cohort. d. Binarized tumour and nodal stages in discovery cohort. e. Effect of class imbalance on separation line. f. Discovery cohort after downsampling to correct for class imbalance. Figure 4. Meta-analysis of the three significant predictors of pCR in the three cohorts shows no significant heterogeneity. Figure 5. Exploratory analyses of variables from copy number (a) and mutations (b) in the discovery cohort. Figure 6. Summary of ML pipeline methods. a. Volcano plot for global limma results. b. Algorithm 1 showing the whole process step-by-step. c. Histogram of p-values. d. Significant genes for 100 iteration. e. Decision making genes using Boruta Figure 7. Alternative RSS models. a. PCA to show technical batch effect in discovery and validation cohort. b-d. ROC curve, score plot and confusion matrix for Gradient boosting machine model on a balanced subset of GSE87211. e-g. ROC curve, score plot and confusion matrix for GBM model on balanced GSE87211 agnostic for pCR. h. AUC on discovery cohort for random 1000 gene sets. i. AUC on balanced validation cohort for random 1000 gene sets. j. AUC on balanced validation cohort prognostic of pCR for random 1000 gene sets. Figure 8. RSS and BRSC are similar across samples without pCR. RSS scores are shown as boxplots split by response in Grampian (a), yT stage in discovery cohort (b) and yT stage in GSE [file mmc2.pptx]

## Slide 1
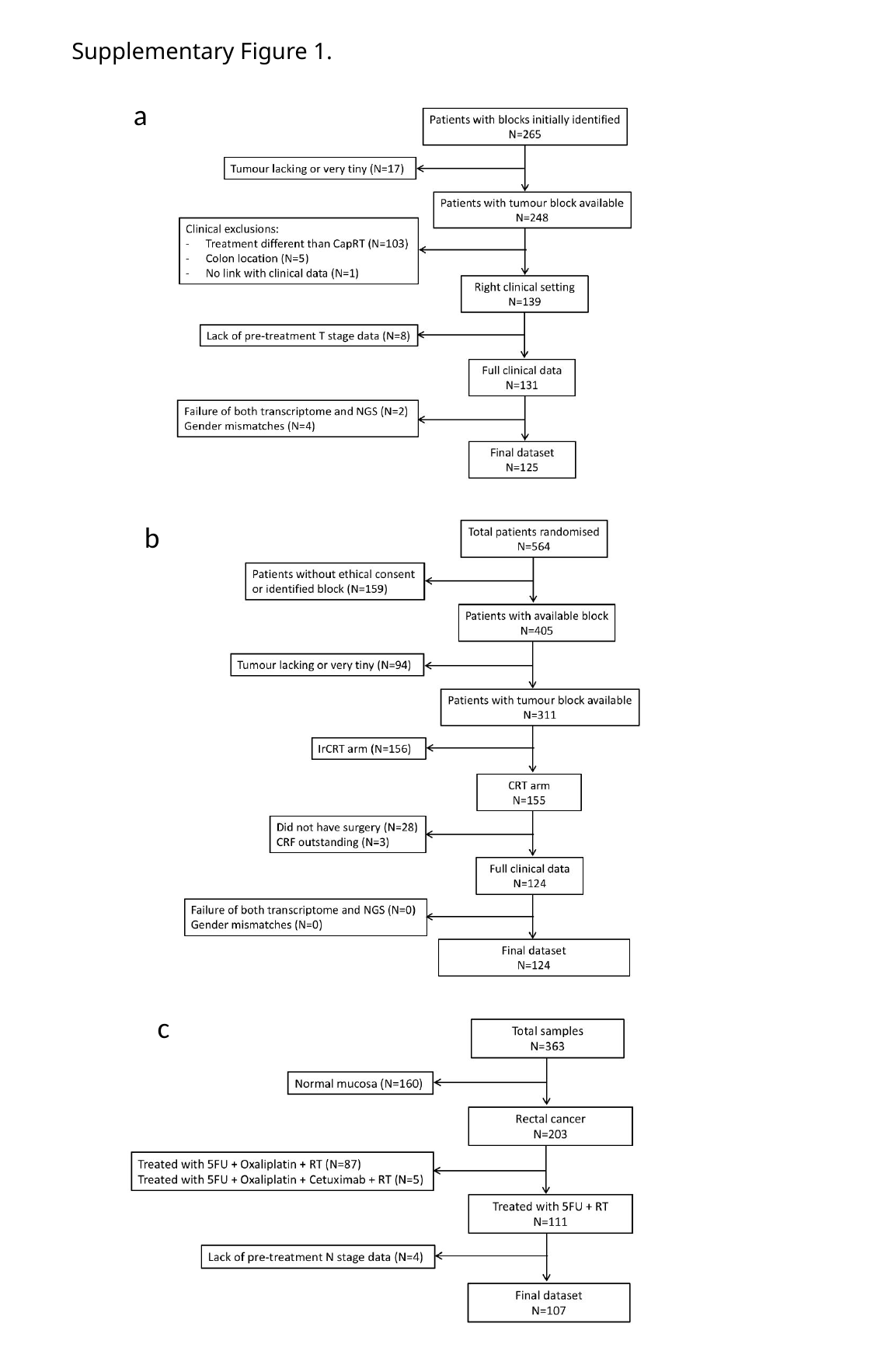

# Supplementary Figure 1.
a
b
c

## Slide 2
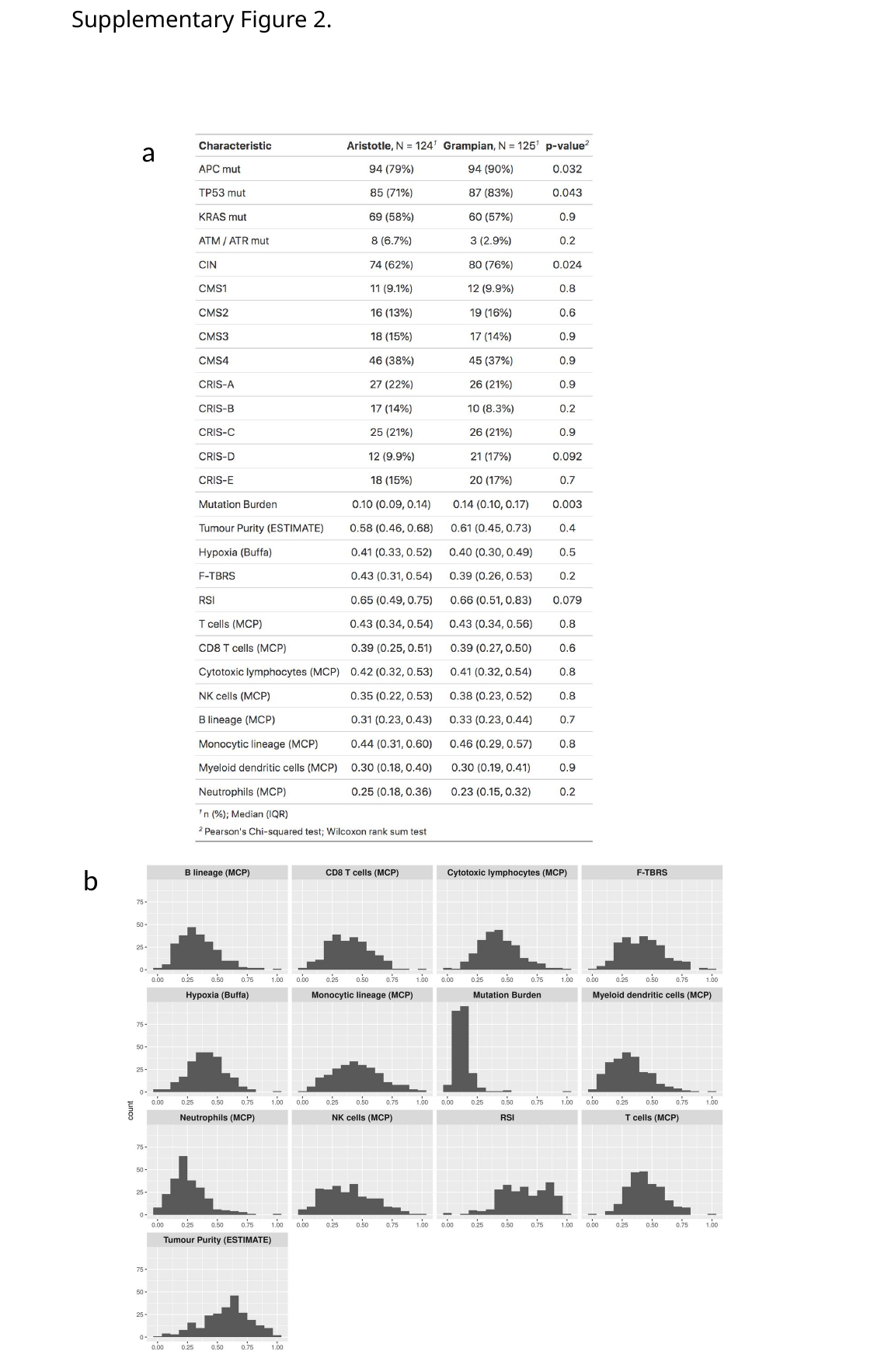

Supplementary Figure 2.
a
b

## Slide 3
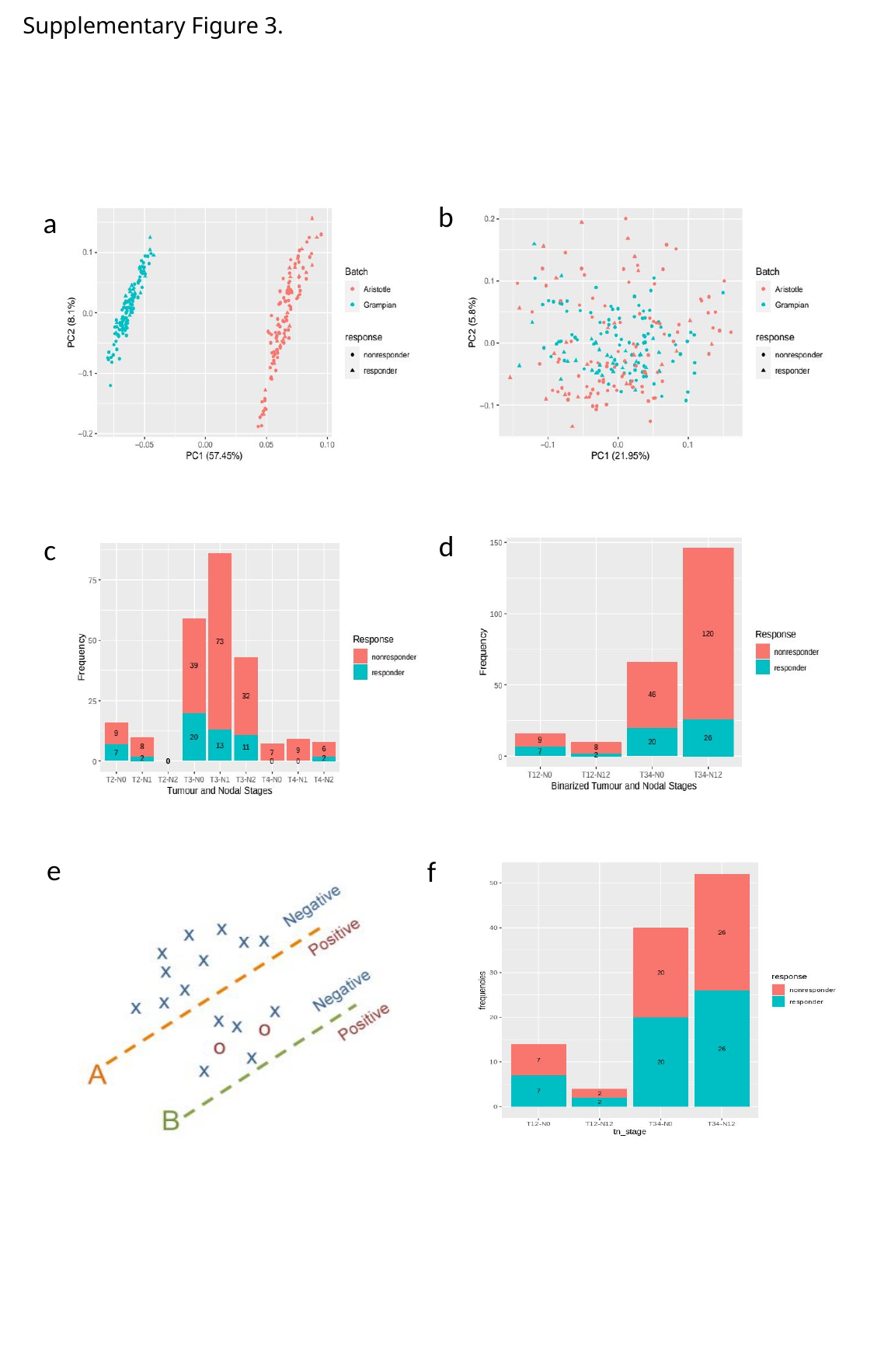

Supplementary Figure 3.
b
a
d
c
e
f

## Slide 4
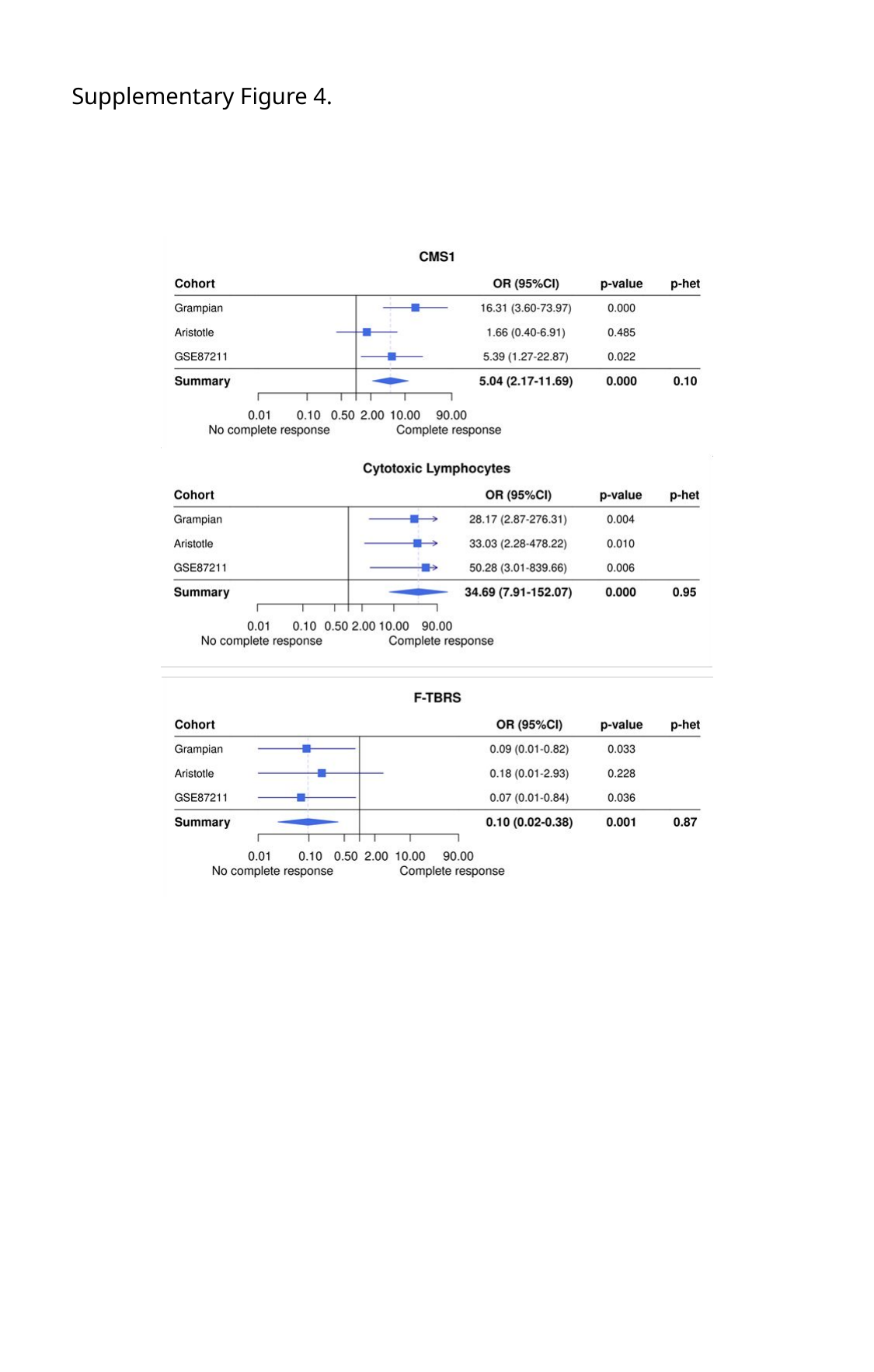

# Supplementary Figure 4.

## Slide 5
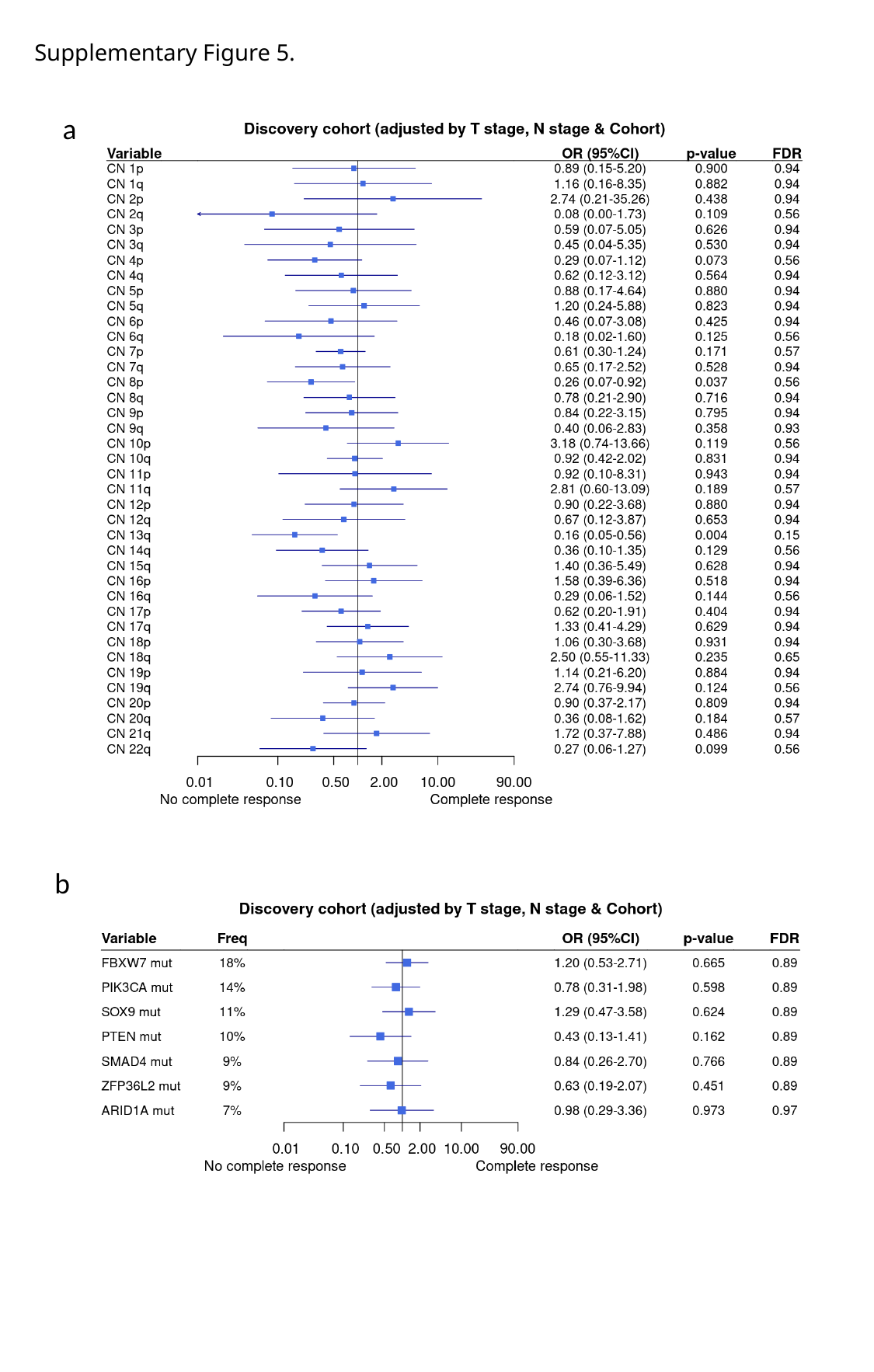

# Supplementary Figure 5.
a
b

## Slide 6
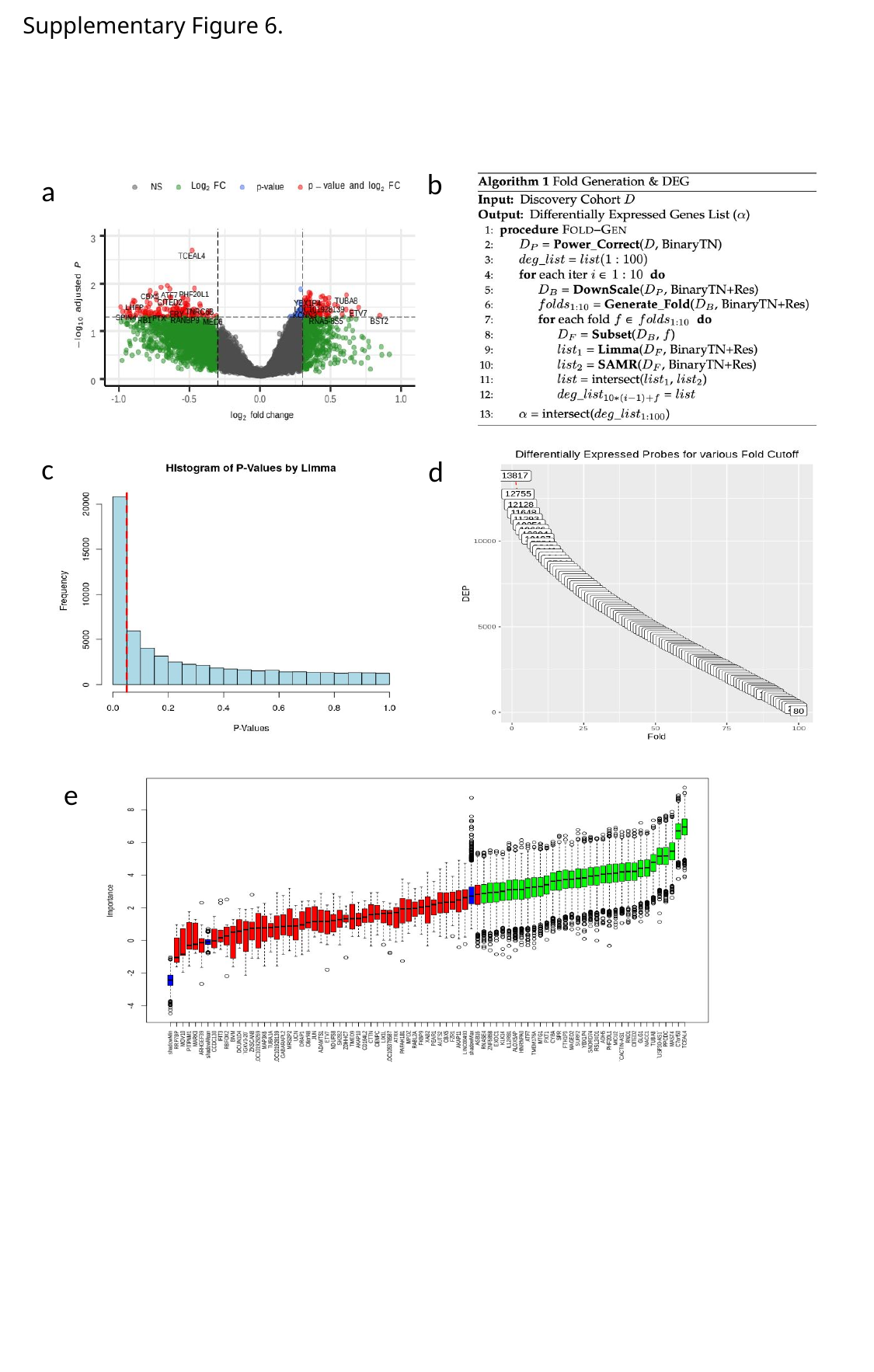

Supplementary Figure 6.
b
a
c
d
e

## Slide 7
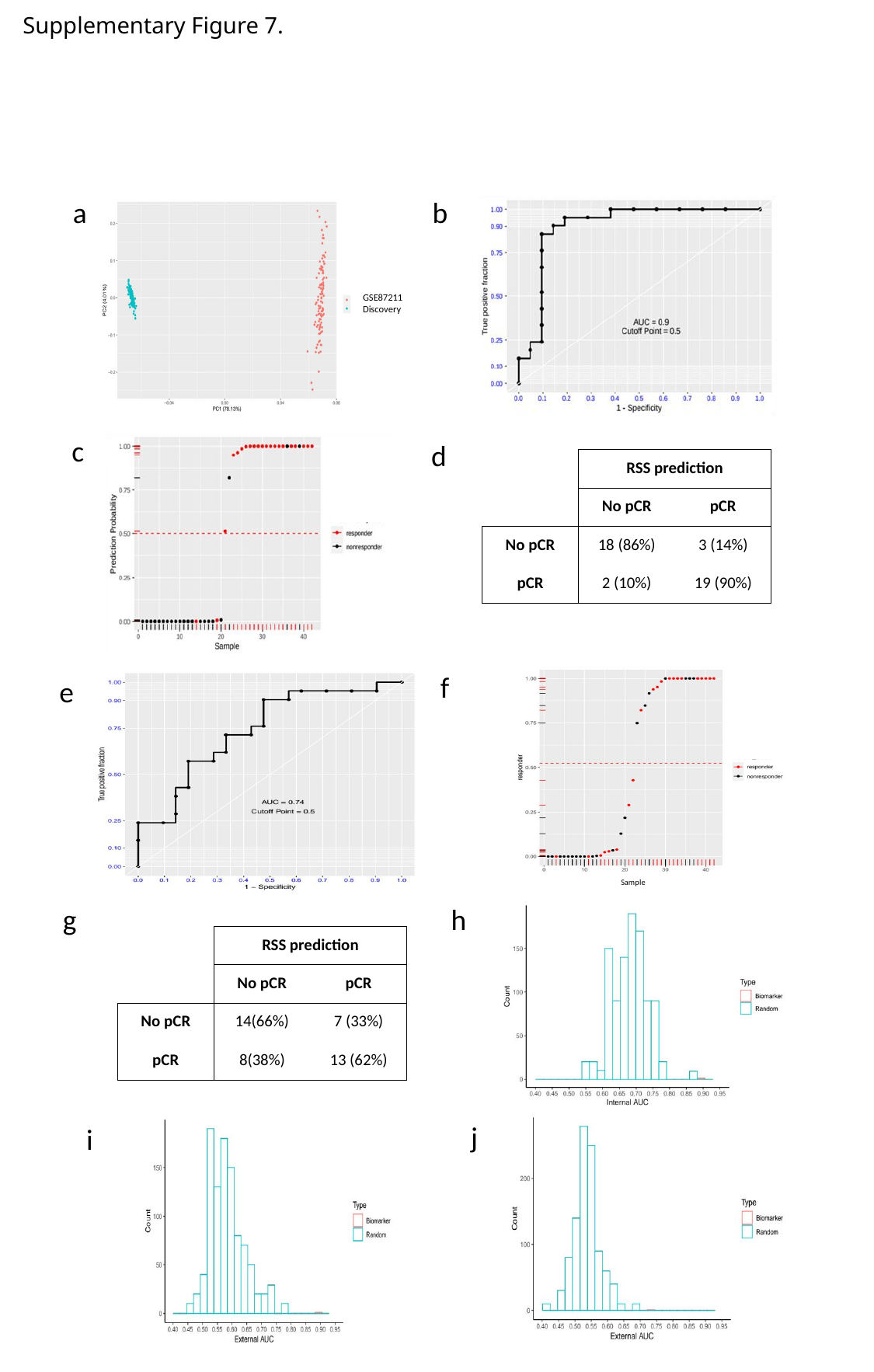

Supplementary Figure 7.
a
b
GSE87211
Discovery
c
d
| | RSS prediction | |
| --- | --- | --- |
| | No pCR | pCR |
| No pCR | 18 (86%) | 3 (14%) |
| pCR | 2 (10%) | 19 (90%) |
f
e
Sample
g
h
| | RSS prediction | |
| --- | --- | --- |
| | No pCR | pCR |
| No pCR | 14(66%) | 7 (33%) |
| pCR | 8(38%) | 13 (62%) |
j
i

## Slide 8
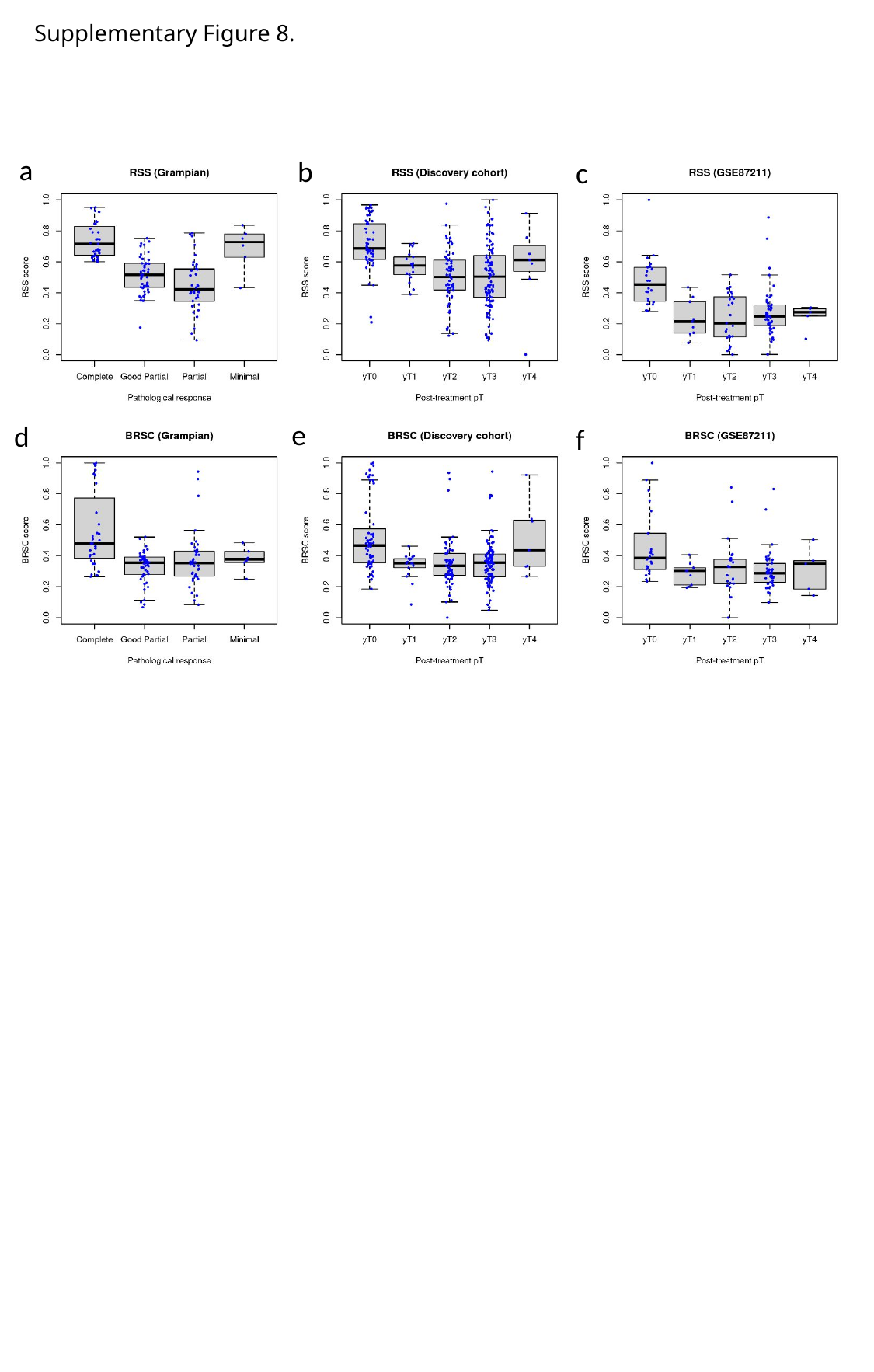

Supplementary Figure 8.
a
b
c
e
d
f

## Slide 9
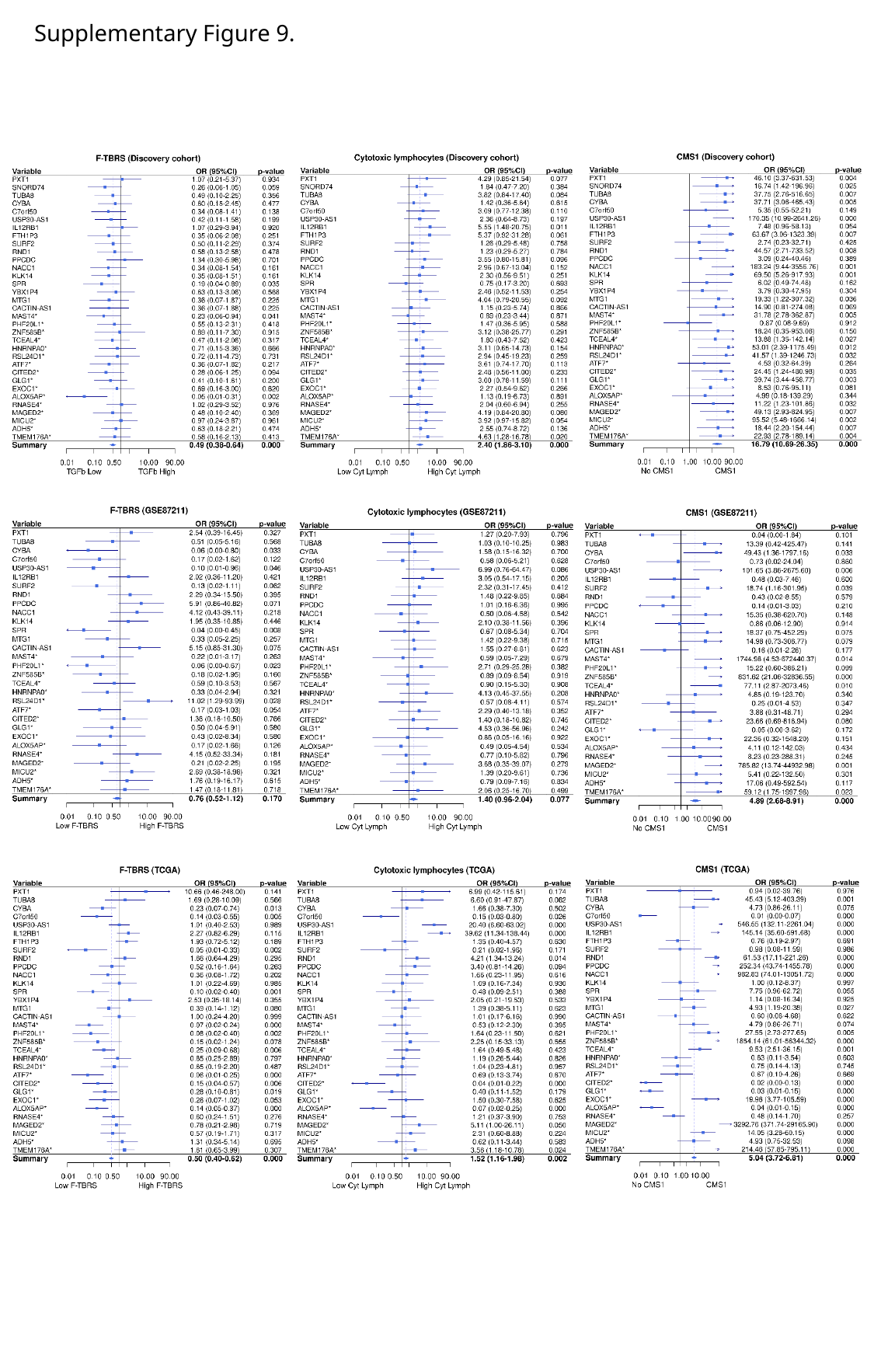

Supplementary Figure 9.

## Slide 10
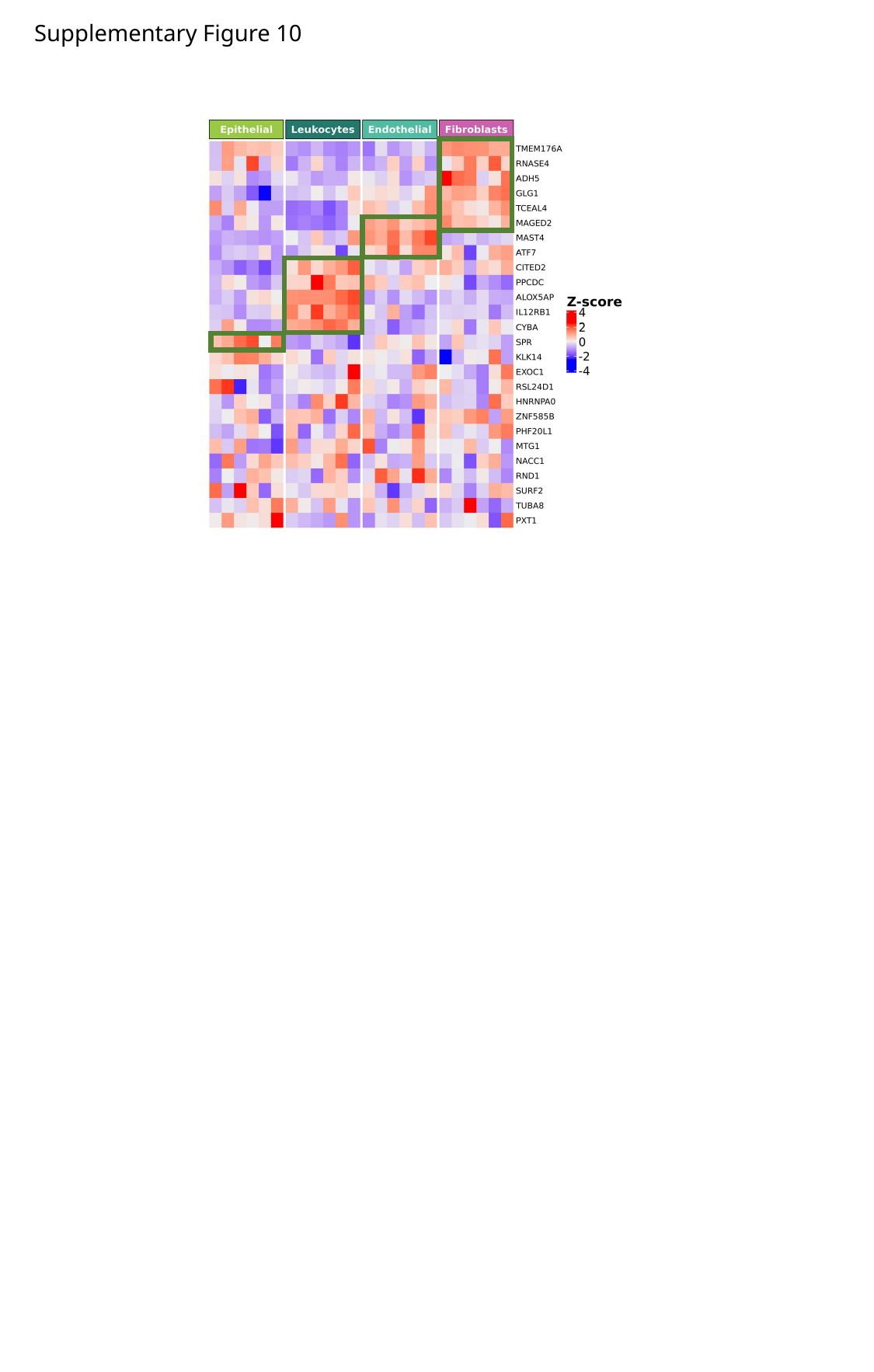

Supplementary Figure 10

## Slide 11
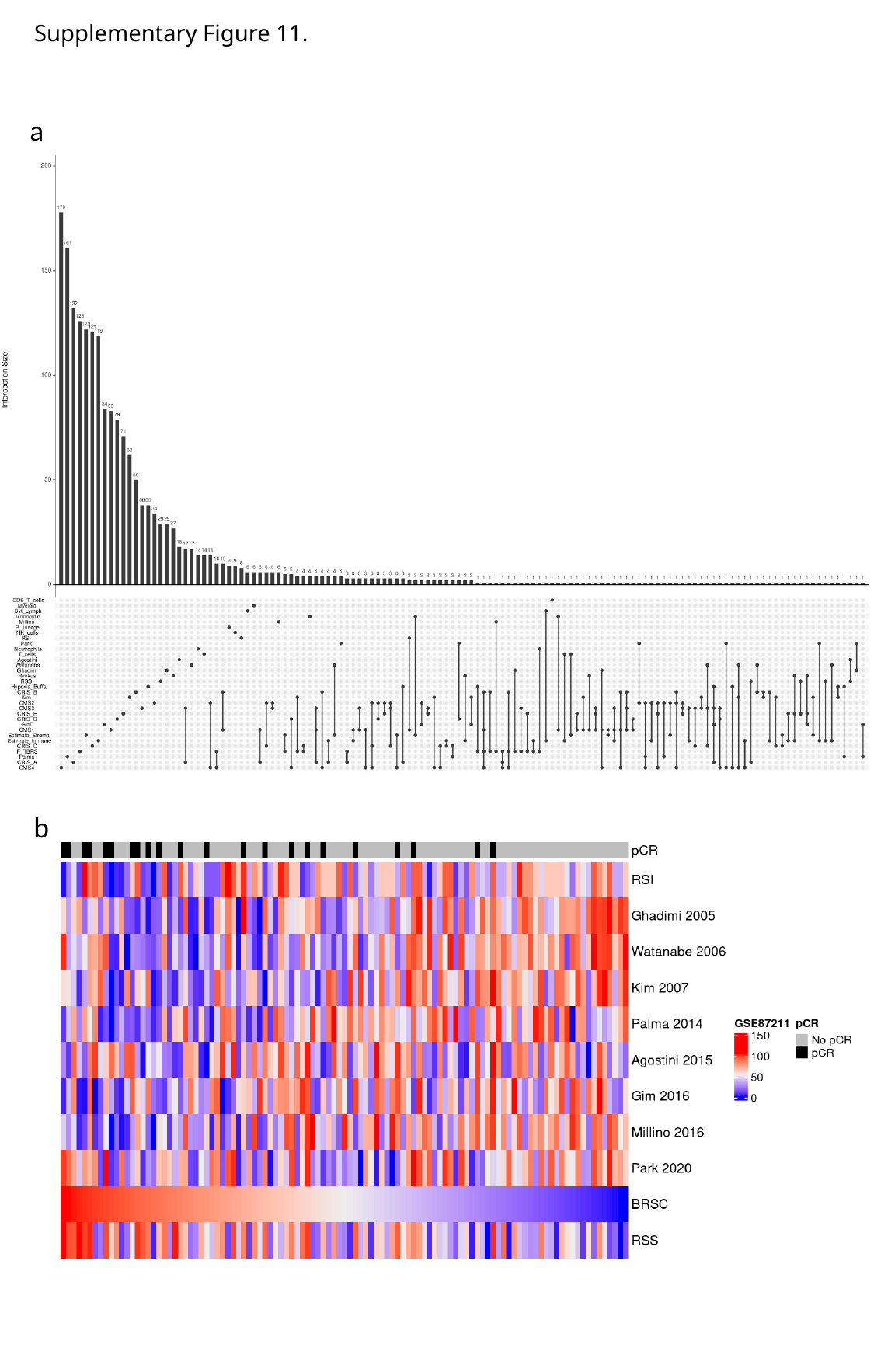

Supplementary Figure 11.
a
b

## Slide 12
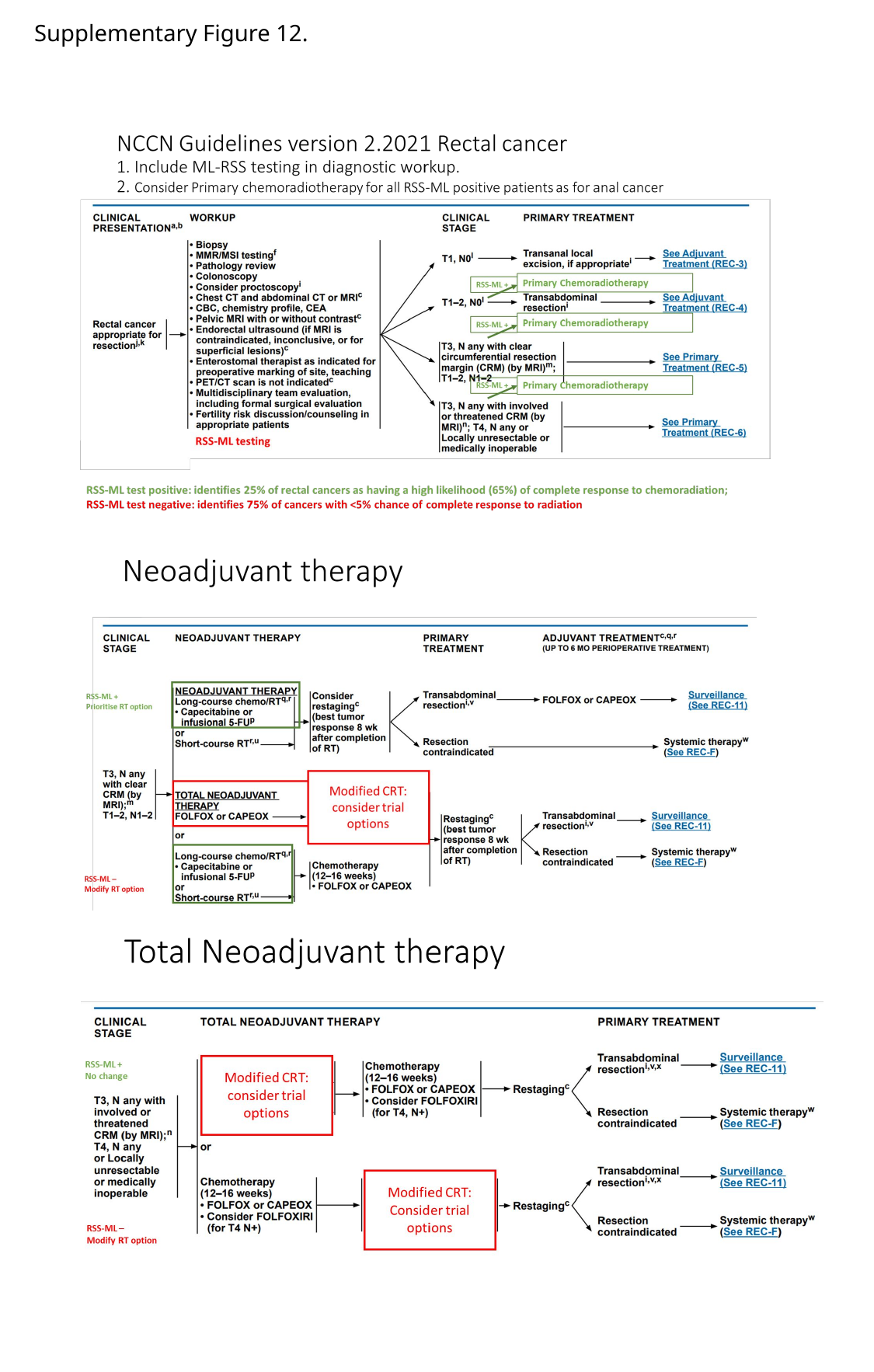

Supplementary Figure 12.
