# Supplementary material for: Identification and validation of a machine learning model of complete response to radiation in rectal cancer reveals immune infiltrate and TGFβ as key predictors
Source: eBioMedicine. 2024 Jul 16;106:105228. doi: 10.1016/j.ebiom.2024.105228 (PMC11663784; doi:10.1016/j.ebiom.2024.105228)
Supplement: Supplementary Methods [file mmc3.docx]

**Supplementary methods**

**Clinical cohorts**

The Grampian cohort comprised a sequential series of patients from the North East of Scotland, including Orkney & Shetland, who had all been treated at Aberdeen Royal Infirmary between 2004 and 2014. Patients had given consent for their sample to be biobanked in the Grampian Biorepository (ref no TR000028, 1/10/2014) with release of linked anonymised clinical data for ethically approved research projects. Patients identified received ‘standard chemoradiotherapy’ (CRT) comprising pelvic irradiation (45-50.4Gy in 25 fractions over 5 weeks) with capecitabine 900mg/m^2^ bd days Monday to Friday, throughout radiotherapy. Clinical data anonymised by S:CORT number was provided comprising demographic data, baseline stage generated from pre-treatment pelvic MRI scans and CT scans TAP, and outcome data. Pathological response was assessed by detailed histopathological assessment of the resection specimen, undertaken 6-12 weeks after CRT, by the same pathologist (GIM) using a four point scale (complete pathological response (pCR), near complete pathological response, partial response, no response).

The Aristotle cohort comprised patients who had entered the ARISTOTLE clinical trial (ISRCTN09351447) between 2011 and 2018 across 75 sites in the United Kingdom, had been randomly allocated to the control arm and in whom biopsies were available for molecular analysis. The Aristotle trial intentionally selected a more advanced subset of rectal cancer than are routinely selected for neoadjuvant CRT. The control arm treatment was identical to standard of care as for the Grampian cohort. Pathological response was assessed centrally according to a pre-specified pathology protocol using the Dworak method (NPW Leeds). Unlocked clinical data were provided by the Aristotle trial team at UCL CRUK CTU.

Both cohorts underwent selection and curation to finally obtain molecular data for 125 and 124 samples from Grampian and Aristotle respectively without any missing data for T/N stage or pCR (Suppl Fig 1A and 1B).

Validation was performed on a publicly available transcriptomic cohort (GSE87211)^1^ that had been profiled with Agilent-026652 Whole Human Genome Microarray 4x44K v2. All analyses in this dataset were performed after completion of analyses in the discovery cohort. All patients were staged as locally advanced (cUICCII/III/IV) and treated according to the CAO/ARO/AIO-94 or the CAO/ARO/AIO-04 trial. Staging included rigid rectoscopy and endorectal ultrasonography, magnetic resonance imaging (MRI) and/or computed tomography as well as patho-histological diagnosis of an adenocarcinoma. Preoperative staging was performed as clinically assessed T-level, lymph node status and distant metastases. Results were unified as clinical UICC stage (UICC). Staging and treatment had either been performed in Gottingen or at five collaborating German Departments of Surgery or Radioncology. Six weeks after the completion of preoperative CRT, curative surgery (total mesorectal excision) was performed. Standardized histopathological work-up resulted in histo-pathological parameters of T-level and lymph node status. For our study we selected samples from tumours treated with 5FU infusion alone and a total irradiation dose of 50.4 Gy (28 x 1.8 Gy) where T and N stage before RT was not missing (Suppl Fig 1C). Tumours showing T0N0 stage after RT treatment were classified as pCRs, otherwise as non-pCRs. Biopsies had been immediately stored on RNAlater, kept at 4C overnight, frozen and stored at -20C.

**DNA Sequencing**

After QC assessment, samples which passed minimum DNA concentration were submitted for library preparation and Illumina sequencing. Library preparations were performed using the Custom SureSelect Library Prep Kit from Agilent Technologies - Part Number 930075. Libraries were submitted for in-solution capture hybridisation using a custom S:CORT colorectal V2 gene bait set, targeting all coding exons of 80 colorectal cancer (CRC) driver genes, 66 regions of recurrent copy number gains/losses, 960 reference SNPs distributed across the genome and 123 MSI regions. DNA amplification was then performed for 8 cycles before sequencing. Sequencing was performed on Illumina HiSeq2000 machines.

The Illumina sequencing data BAM files were transferred to a dedicated variant calling pipeline developed by the Cancer Group Project (CGP) team for sequence alignment and data analyses. Variant calling algorithms were first run on the data for identification of base insertion/deletions and substitutions in the data using Pindel and Caveman C CGP algorithms. The data was then flagged for variants present only in the list of the cancer genes of the custom bait design as well as for variants seen in germline, FFPE normal samples to remove sequencing artefacts.

Copy number at low resolution was analysed with CNVkit^2^ adjusting by tumour purity as derived from RNA expression with the Estimate R package^3^ where available. Copy number estimates were summarised as loss (cn ≤ -1), neutral (cn = 0) or gain (cn ≥ 1). Samples showing neutral copy number calls in >20% of the length of all chromosomes combined were classified negative for Chromosomal Instability (CIN), otherwise as positive^4^. Copy number calls at the chromosome arm level were done by making the sum in length of the three summarised copy number estimates and selecting the longest one across each arm.

Tumours showing more than two mutations in 123 MSI markers within the panel were classified as MSI, otherwise as microsatellite stable.

**RNA Profiling of FFPE tissue**

To harness the power of large archived clinically annotated cohorts of FFPE tumour blocks, we utilized FFPE-specific IVT reagents and the Almac Xcel Array. These arrays have probe designs focused on the extreme 3' of mRNA (Affymetrix Almac Xcel Array), as these regions are more stable in FFPE tissue. FFPE sections were dewaxed and macrodissected following our S:CORT standard operation procedure. The Roche High Pure RNA Paraffin Kit instructions for use (Version 12) were followed for RNA extractions. QC metrics relating to monitor image quality, IVT, hybridisation to the array and RNA degradation were assessed prior to uploading to the Oxford server, where further QC was performed. Probeset and gene level data was generated and loaded into the MYSQL database at Oxford. Gene level data had been generated by getting the median of all probesets in each gene. Initially Grampian and Aristotle data were analysed separately for analyses within each cohort. For analyses combining them both we used the combat function from the sva R (Version 3.30) package to correct for batch effects by cohort that were identified by PCA. Although all samples had been identically processed in the same labs, they had been run about two years apart. Grampian was used as a reference with clinical variable pCR in the argument mod in order to preserve variance related to the response. The single set was called ‘discovery cohort’.

**Data Integration QC:** **Clinical vs Molecular Gender**

Gender was used to check for potential sample switching by checking molecular gender in the multiomic platforms. For copy number the estimates of X and Y chromosomes were plotted. From RNA expression, gender was checked using the levels of the XIST gene and also a 3-Dimensional PCA graph on an Almac list of gender probesets. Rare identified errors had their source identified and were corrected or excluded.

**Bioinformatics and hypothesis-based analysis**

A list of pre-defined, hypothesis-based candidates for response to RT considering the molecular data available was built by an expert panel of researchers (Suppl Table 1). This covered both molecular profiles strongly linked with response to RT in the literature and relevant classifiers for CRC based on strong, distinct biology usually associated with outcome. Candidates were selected based on broad tumour biology rather than focusing on rectal cancer. Only gene sets from bona fide signatures shown to reliably score specific biological features at the single sample level were considered, as opposed to gene sets such as MSigDB initially built for comparison techniques like GSEA. Overlapping biology was avoided. Binary variables with frequency <5% in Grampian and Aristotle combined were discarded due to suboptimal statistical power. Accordingly, driver mutations in ATM and ATR were combined as individually they were too infrequent. Hypermutation was not selected as only 2 MSI and 2 POLE cases were identified. Such low frequency is expected in the clinical setting of rectal cancer^5^.

RNA-based signature profiles were derived using the R packages originally provided or alternatively by replicating the same methods as in their original reports (Suppl Table 1). For CMS, since the profiles derived with the original CMSclassifier^6^ (version 1.0.0) are strongly associated with the presence of nonmalignant stromal regions^7^, which may be underrepresented or even fully missing in several biopsies, the alternative CMScaller (version 2.0.1) method designed for preclinical models (e.g. with lack of tumour microenvironment) was applied^8^.

All variables were scaled from 0 to 1 to make them statistically comparable. Then logistic regression models for each candidate were built based on presence of pCR adjusted by the clinical confounders of T and N stage as continuous variables which were determined from the pretreatment MRI assessment (i.e. at the time of clinical decision). The binary variable cohort was also used for adjustment of all analyses using Grampian and Aristotle combined (discovery cohort). Variables showing p<0.1 in the adjusted univariable analysis were selected to build a multivariable model combining them all, adjusting for continuous T and N stage. Then backward stepwise regression was performed by filtering the variable with the highest non-significant p-value to obtain a reduced final model with variables showing additive, independent prediction ability at p<0.05. When more than one variable was found in the final model, this was summarised as a composite variable per sample by adding the scaled values of each molecular component associated with pCR and then subtracting the values from molecular components associated with lack of pCR. Goodness of fit of the model was done by likelihood ratio test comparing models of the geneset, T stage and N stage with a model with only T and N stage. Meta-analysis of the three independent cohorts for each of three final variables was performed to show evidence of similar signals. A fixed effect model was built where a p-value for heterogeneity was also computed where high values suggest homogeneity and p<0.05 higher levels of heterogeneity than chance.

**Machine learning algorithms and methodology**

In a second, hypothesis free analysis, our aim was to build a Machine Learning (ML) model to predict pCR after RT in rectal cancer patients from transcriptomic data. Twelve different ML algorithms were implemented in a multi-step pipeline: Quality check, Pre-processing, Class Balancing, differentially expressed genes (DEG) selection, Decision making genes (DMG) selection, Train­ing to build a model (ML) from the discovery cohort. Apart from quality check and pre-processing, which were performed on the whole dataset, all other steps were performed in cross-validation. These steps and the cross-validation design are detailed below. Some of the pre-processing details can also be found in the RNA profiling section above. We strictly follow the FDA guidelines^9^ (<https://www.fda.gov/media/122535/download?attachment)> throughout the process of model training and validation. We have developed a 'locked' model, which is inherently more reliable compared to a learning model. According to the FDA, a 'locked' algorithm is one that consistently produces identical results when given the same input and remains unchanged with repeated use.

*Quality Check and Pre-processing for the ML pipeline:*

238 samples with an unbalanced T and N stage were used from the discovery cohort, 121 from Grampian and 117 from Aristotle (where 4 cases were not used due to missing clinical data at the time). The T stage was dominated by T3 samples (188 out of 238). There was only one T1 sample so we combined it with T2 samples. The N stage was more balanced: N0 (82), N1 (105) and N2 (51).

The discovery set having 238 samples posed several challenges like batch effect, power correction, class imbalance, and causal inference. During the pre-processing step we tried to tackle these challenges with traditional as well as innovative solutions.

*Pre-processing: Batch Effect*

The Grampian and Aristotle cohort expression was extracted in two different clinical settings with varying protocols and methodologies. Therefore, we used PCA (Principal Component Analysis) to check any batch effect between the Grampian and Aristotle cohorts. A major batch effect was found between the two cohorts making them land on different scale (Supp Fig 5A). Hence, scaling using batch correction tools becomes a necessity. To perform batch correction we have chosen ComBat as it is the most broadly endorsed by the transcriptomics community (Supp Fig 5B)^10^. Further discussion on batch effect correction is also provided in the validation section.

*Pre-Processing: Binarization of clinical staging*

A discovery set without enough power is not suitable for further analysis. Therefore, power correction of the discovery cohort is one of the most important steps. The response group has shown enough power with 55 responders (pCRs) and 183 non-responders (not-pCRs). But the T and N stages have some issues due to a few categories with no samples (Supp Fig 5C). After a thorough discussion with clinicians, we decided to binarise the T stage (1,2 and 3,4) as well as the N stage (0 and 1,2) (Supp Fig 5D) to avoid “NA” results during model training.

*First step in cross-validation: Class Imbalance*

Imbalanced classes are one of the common problems in biological datasets where there are a disproportionate ratio of observations in each class. Supp Fig 5E shows an example where class imbalance can be a cause of a false cutoff point. Most of the ML models target to minimize the error for cutoff point. Therefore, they will choose point B as cutoff with error of 2 instead of actual cutoff point A with error of 5 during training. The discovery set showed class imbalance with 55 pCRs and 183 non-pCRs. Therefore, we used sampling techniques to balance the classes. We tried to avoid any sampling method which add hypothetical samples because it could be harder to defend hypothetical patient data. Hence, we used downsampling to balance the power between responders and non-responders (Fig 5F).

*Second step in cross-validation: Selection of Differentially Expressed Genes*

After sampling, a clean CRC patient dataset (well-powered, class balanced, no batch effect) was available for differentially expressed genes (DEG) analysis. Specifically, we used Limma (Version 3.46) and SAMR (Version 3.0) with resampling to find differentially expressed genes. We modified standard Limma and SAMR analysis to account for cancer stage and outliers. Cancer stage could be introduced as a confounding factor along with pCR to handle the causal inference by staging. However, this could generate problem with different power in different stages sub-groups of the discovery cohort. Accordingly, we chose to modify the Limma/SAMR step by including the different stage data based on their power ratio in the whole discovery cohort. In this way, Limma/SAMR finds statistics for each gene in all stages and calculates their overall statistics based on the power ratio for each stage. A volcano plot shows significantly expressed genes with p-value *<* 0.05 and fold change *>* 1.5 using this approach with Limma (Suppl Fig 6A).

Another main issue was the effect of outlier samples. These could be real biological outliers as well as the sample mis-read due to technical or human error. Taking them out completely might miss real biology, including them may misguide the analysis. We addressed this by resampling. Specifically, in each resampling iteration we performed Limma and SAMR analysis on 90% of the samples and derived differentially expressed genes. We then selected the genes significant across all iterations.

Algorithm 1 shows the whole process step-by-step (Suppl Fig 6B). The first step was to power correct discovery cohort by binarizing T and N stage so that every category have at least a few sample (line 2). Then, a list of length 100 is generated to store statistics for differentially expressed genes in 10 iterations where every iteration has 10 folds. Initially, for every iteration, the discovery cohort is downscaled to have 55 pCRs and 55 random non-pCRs (lines 4-5). Now, in every fold of an iteration, 49 random pCRs and 49 non-pCRs are used to find differentially expressed genes using Limma and SAMR (lines 7-12). At last, an average is taken for all genes to calculate the overall statistics. A gene is considered differentially expressed if it comes out significant in every resampled dataset. The p-value histogram of final statistics shows the possibility of a strong biomarker (Supp Fig 6C). We found 80 genes that were significant in all 100 computations after running our personalized Limma on the discovery cohort (Fig 6D).

*Third step: Decision Making Genes*

We found 80 significant genes from DEG analysis on the discovery cohort. Generally, bioinformaticians use significant gene lists as a biomarker and use them to predict the response of a CRC patient for a given therapy. However, some significant genes can be the reason for noise instead of contributing to prediction. Therefore, instead of using the significant genes from DEG analysis directly as a biomarker, we used state of the art ML methods to optimize our significant gene list and called them decision-making genes (DMG). We also used Boruta (Version 7.0.0), a random forest-based R package for feature selection with ML models lacking stringent feature selection capabilities (Supp Fig 6E). Boruta uses an iterative approach to find importance of each feature and drop the least important features. Therefore, it helps the ML models to avoid unnecessary noise due to less important features. Models trained after feature selection are shown with prefix F_ (Fig 3B).

Instead of choosing a ML method a priori, we allowed our discovery cohort data to suggest the best performing ML. Therefore, we used 6 different ML methods (from R caret package version 6.0.84) namely elastic net, logistic regression, support vector machines, neural networks, random forest and gradient boosting machines on our discovery cohort with nearly the same criteria and evaluated them based on leave-one-out and k-fold cross-validation. These 6 ML methods were used to perform 12 ML models (with and without feature selection).

All ML methods are fine-tuned using grid search on hyperparameters. Elastic Net uses a grid of length 100 for lambda 0.001 to 1000 and repeated cross-validation with 10 repeats having length 10 to minimize mean squared error. Logistic regression uses the same repeated cross-validation with a binomial kernel. Support vector machines use a grid of length 100 for C (Penalty parameter of the error term) 0 to 5 and the same repeated cross-validation. Neural networks use two hyperparameters with repeated cross-validation. The grid has a two-dimensional matrix with the size (number of units in hidden layer) from 1 to 10 with a change of 1 and decay (regularization parameter) from 0 to 0.5 with a change of 0.1. The tuneRF function Caret function is used to fine-tune random forest. Gradient boosting machines use repeated cross-validation with a grid of four hyperparameters (interaction depth 1 to 10 with a change of 1, number of trees 50 to 1500 with a change of 50, shrinkage 0.1 to 100 with multiply change of 10, number of minobsinnode 10 & 20). An implementation of these models with hyperparameters is available on GitHub (https://github.com/sanjaysinghrathi/SCORT-ML-Pipeline).

The ML method with the best accuracy and mean precision was chosen to validate on the publicly available external cohort. The features in the selected optimal ML model were considered as DMG (Fig 3B).

*Validation*

A publicly available cohort GSE87211^1^ was used to validate our locked ML model. This validation cohort was pre-processed to remove batch effects and class imbalance.

The combat function was used to remove the batch effect between discovery and validation cohorts^10^. Generally, it is not a good practice to merge/preprocess validation cohorts with discovery cohorts. But clearly, we are not yet operating in clinical settings. In those settings, every sample will be analyzed in the same lab with the same methodology and obviate the need for preprocessing. In the current scenario, the development of any classifier relies on retrospective datasets which were accrued at different times and of course have different analytical protocols and methodologies for transcriptomics analysis. In our specific case, the differences in the gene expression distributions between the datasets is well illustrated (Supplementary Figure 7). The PCA plot in Fig 7A for the discovery and validation cohort shows them on two different planes. It would be impossible to train a model on one plane and test it on another with such cohort-based batch effect, without accounting for the batch effect.  Therefore, batch correction becomes a necessity

On the other hand, batch correction has an underlying issue which is the possible distortion and degradation of the biological signal. This is unavoidable to some extent, as any batch correction would distort the data to minimize the distance between datasets. However, to limit the distortion of the biological signal, ComBat^10^ and other batch correction methods, require statement of the variables which should be used for batch correction, and other variables which should not be used.

The purpose of this process is to remove the variances due to cohort while preserving any biological variance due to pCR to treatment. This allows to separate the biological signal from the batch effect, and correct the latter while minimizing distortion of the former. In conclusion, while the use of pCR information during scaling/mapping should be depreciated in a clinical setting, simple scaling can result in the flattening of signal due to pCR in a highly imbalanced cohort like GSE87211.

Accordingly, the discovery cohort was used as reference and pCR status in the argument mod as previously done to merge Grampian and Aristotle. However, to ensure the relevance of our classifier for prospective application, we also asked if our locked ML model was still predictive in a validation fully agnostic of pCR data during batch correction. Thus, an additional GSE87211 transcriptome was also built with the combat function^10^ but using binarised pretreatment T/N stage in the mod argument rather than pCR, mindful that these are different signals and so the resulting data would not have preserved the integral variance by pCR status. This further validation provides a “worst-case scenario” for the AUC with respect to a clinical setting, where protocols for transcriptomics are standardized and batch correction is not needed.

We used downsampling to prepare a balanced validation cohort. The downsampling process selected 21 pCRs and 21 random non-pCRs from a set of 107 samples in the validation cohort. Finally, the F_GBM model was tested on both the complete and balanced validation cohort to derive p-value, sensitivity, specificity and area under curve (Fig 7B-7G).

Finally, a randomized permutation experiment was done to further test the significance of our geneset (Fig 7H-7J). In this experiment, an empirical null was generated by randomly picking 1000 gene sets (each containing 33genes).

We have followed TRIPOD guidelines for prediction model and validation (Suppl Table 5)

**Gene Set Enrichment Analysis (GSEA)**

To better understand which biological pathways may be associated with the ML signature and make a fair comparison with the ones associated with radiation response, we used GSEA. DEA using gene entrez id data was run with Limma in GSE87211 for the binary prediction of the ML model comparing predicted pCRs against predicted to be not-pCRs. The same analysis was also performed comparing actual pCR against not-pCR. All 50 hallmark gene sets from Molecular Signature Database (MSigDB v7.5)^11^ were run with an adjusted p-value of 0.01.

**Comparison between candidate and ML models**

To evaluate the biological relevance of the final ML model, every gene from the ML model was individually analysed by univariable linear regression as the dependent variable using one of the molecular variables from the hypothesis-based model as the independent variable. For this analysis the scaled values from genes associated with lack of pCR were reversed so that the association could be compared with genes associated with pCR. This allowed us to meta-analyse the regression results from all genes of each analysis using a fixed effect model from where OR, 95% CI, p-value and heterogeneity p-value were computed, where the latter denotes higher levels of heterogeneity than chance when <0.05. This analysis is agnostic to treatment outcome so it was also run on a large third cohort of TCGA (COAD and READ combined). RNAseq transcriptomic data was retrieved with the R package TCGAbiolinks (version 2.20.1)^12^. GSE87211 and TCGA transcriptomes were processed similarly to the discovery cohort to derive relevant RNA signatures.

**Expression by cell type**

It is well recognised that the transcriptome is strongly influenced by presence of stromal and other cell types^13^ so we aimed at testing the potential influence of such effect on our ML signature. A heatmap was generated using confoundR (<https://confoundr.qub.ac.uk/>) for the cohort GSE39396. This dataset comprises four different cell type populations separated by FACS from 6 CRC cases: epithelial cells (EPCAM+), leukocytes (CD45+), fibroblasts (FAP+) and endothelial cells (CD31+).

**Analyses of previously published signatures**

We reviewed the literature to identify previously published reports of similar RNA expression signatures for prediction of response to RT in rectal cancer (Supp Table 1). These were compared with our models after they were generated. Genes for each identified signature were annotated together with their positive or negative association to RT prediction. Scores for each signature in each sample in GSE87211 were generated by getting the RNA expression mean of the positive and negative genes separately, and then subtracting them. The signature from Rimkus et al^14^ had not published directionality of the genes so it was not scored. The signature RSI was also included but scored as originally reported^15^ and used in our candidate analysis. The scores within each signature were then scaled from 0 to 1. The same method was applied for our predictive models to get comparable signature scores (as opposed to the predictive scores used in our ML methods). These signatures scores were used in analyses for correlation, regression and distribution across response/T stage.

A heatmap of all the signatures was run sorting samples by the ML signature. Since the distributions of the signatures were very variable and not comparable, samples were ranked according to each signature score.

Overlap of gene content was performed using entrez ids which conform unique identifiers for proper comparison. Entrez ids of relevant signatures were used when these had been published, otherwise, published gene symbols were converted into entrez gene ids using biomaRt R package (version 2.48.3). If gene symbols were mapped to more than one entrez gene, all of them were kept. If no entrez gene was identified, it was not present in the final set. Accordingly, in some signatures the total number of genes may differ compared to the originally reported.

**Analysis of additional mutations and copy number**

Genes showing driver mutations at frequency >5% in Grampian and Aristotle combined that had not been used in the hypothesis-driven analysis were selected. All available copy number by chromosome arm was analysed as a continuous variable scaled from 0 to 1 (0 for loss, 0.5 for neutral, 1 for gain). Logistic regression models for each candidate were built based on presence of pCR and adjusted by pretreatment T and N stage. As these variables were not candidates, significance was called after correcting for FDR at <0.05. Candidates in the hypothesis-driven analysis had not been corrected for multiple testing as applying FDR under this situation might increase the chances of losing true positives rather than decreasing false positives because variables were not random but strongly selected.

**References**

1. Hu Y, Gaedcke J, Emons G, et al. Colorectal cancer susceptibility loci as predictive markers of rectal cancer prognosis after surgery. *Genes Chromosomes Cancer* 2018; **57**(3): 140-9.

2. Talevich E, Shain AH, Botton T, Bastian BC. CNVkit: Genome-Wide Copy Number Detection and Visualization from Targeted DNA Sequencing. *PLoS Comput Biol* 2016; **12**(4): e1004873.

3. Yoshihara K, Shahmoradgoli M, Martinez E, et al. Inferring tumour purity and stromal and immune cell admixture from expression data. *Nat Commun* 2013; **4**: 2612.

4. Burrell RA, McClelland SE, Endesfelder D, et al. Replication stress links structural and numerical cancer chromosomal instability. *Nature* 2013; **494**(7438): 492-6.

5. Papke DJ, Jr., Yurgelun MB, Noffsinger AE, Turner KO, Genta RM, Redston M. Prevalence of Mismatch-Repair Deficiency in Rectal Adenocarcinomas. *N Engl J Med* 2022; **387**(18): 1714-6.

6. Guinney J, Dienstmann R, Wang X, et al. The consensus molecular subtypes of colorectal cancer. *Nat Med* 2015; **21**(11): 1350-6.

7. Dunne PD, McArt DG, Bradley CA, et al. Challenging the Cancer Molecular Stratification Dogma: Intratumoral Heterogeneity Undermines Consensus Molecular Subtypes and Potential Diagnostic Value in Colorectal Cancer. *Clin Cancer Res* 2016; **22**(16): 4095-104.

8. Sveen A, Bruun J, Eide PW, et al. Colorectal Cancer Consensus Molecular Subtypes Translated to Preclinical Models Uncover Potentially Targetable Cancer Cell Dependencies. *Clin Cancer Res* 2018; **24**(4): 794-806.

9. Proposed regulatory framework for modifications to artificial intelligence/machine learning (AI/ML)-based software as a medical device (SaMD). [*https://wwwfdagov/media/122535/download?attachment*](https://wwwfdagov/media/122535/download?attachment) 2021.

10. Zhang Y, Parmigiani G, Johnson WE. ComBat-seq: batch effect adjustment for RNA-seq count data. *NAR Genom Bioinform* 2020; **2**(3): lqaa078.

11. Liberzon A, Birger C, Thorvaldsdottir H, Ghandi M, Mesirov JP, Tamayo P. The Molecular Signatures Database (MSigDB) hallmark gene set collection. *Cell Syst* 2015; **1**(6): 417-25.

12. Colaprico A, Silva TC, Olsen C, et al. TCGAbiolinks: an R/Bioconductor package for integrative analysis of TCGA data. *Nucleic Acids Res* 2016; **44**(8): e71.

13. Fisher NC, Byrne RM, Leslie H, et al. Biological Misinterpretation of Transcriptional Signatures in Tumor Samples Can Unknowingly Undermine Mechanistic Understanding and Faithful Alignment with Preclinical Data. *Clin Cancer Res* 2022; **28**(18): 4056-69.

14. Rimkus C, Friederichs J, Boulesteix AL, et al. Microarray-based prediction of tumor response to neoadjuvant radiochemotherapy of patients with locally advanced rectal cancer. *Clin Gastroenterol Hepatol* 2008; **6**(1): 53-61.

15. Eschrich S, Zhang H, Zhao H, et al. Systems biology modeling of the radiation sensitivity network: a biomarker discovery platform. *Int J Radiat Oncol Biol Phys* 2009; **75**(2): 497-505.
